# A Systematic Review of Obstetric Mistreatment Among Women Living With HIV

**DOI:** 10.1111/nhs.70323

**Published:** 2026-03-31

**Authors:** Joy Edeh, Oluwaseun Badru, Roba Alwasila, Ezinwa Anyanwu, Oluwafemi Adeagbo

**Affiliations:** ^1^ Department of Epidemiology The University of Iowa Iowa City Iowa USA; ^2^ Department of Community and Behavioral Health The University of Iowa Iowa City Iowa USA; ^3^ Ubiquitous Educational Consult Limited Akoka Lagos State Nigeria; ^4^ The University of Memphis Memphis Tennessee USA

**Keywords:** HIV‐positive women, obstetric mistreatment, obstetric violence, women living with HIV

## Abstract

Several studies have established that women living with HIV frequently encounter mistreatment when accessing maternal healthcare. Previous reviews on obstetric mistreatment have largely focused on the general population, leaving an important research gap to fill. As such, we synthesized evidence of obstetric mistreatment among women living with HIV. Six databases were searched in January 2025: CINAHL Plus, Embase, PubMed, Scopus, Web of Science, and Google Scholar. Of the 4652 articles assessed, 23 studies were included. There was evidence of varying proportions of non‐consented care, lack of privacy, non‐dignified care, abandonment and neglect, forced sterilization, stigma and discrimination, physical abuse, non‐confidential care, and lack of supportive care from maternal healthcare providers. Our qualitative findings revealed experiences of disrespect and abuse, stigma and discrimination, non‐dignified care, and forced sterilization. There is substantial evidence that women living with HIV continue to experience diverse forms of mistreatment in maternal care, particularly in Sub‐Saharan Africa. Our findings highlight the urgent need for systemic and structural reforms in maternal healthcare settings to improve the experiences of women living with HIV.

## Key Points

- Most studies on obstetric mistreatment among women living with HIV are from Sub‐Saharan Africa, with more studies needed from other regions.
- While there are many forms of obstetric mistreatment, forced sterilization was the most concerning because it sometimes happens without women's consent.
- Health care professionals need urgent training and retraining on sexual and reproductive health care for women living with HIV.

## 1 Introduction

According to the Joint United Nations Program on HIV/AIDS (UNAIDS), 44% of new HIV infections occurred in women and girls globally in 2023 (Global HIV and AIDS Statistics 2024). Approximately 4000 young women and girls between the ages of 15 and 24 globally were infected with HIV every week in 2023 (Global HIV and AIDS Statistics 2024). Sub‐Saharan Africa bears the most significant burden (Lu et al. 2025), with an occurrence of about 62% of new HIV infections in women and girls of all age groups in 2023 (Global HIV and AIDS Statistics 2024; Lu et al. 2025). The high HIV seroprevalence in young girls and women calls for a greater understanding of the socio‐cultural and structural influences contributing to these disparities.

The mistreatment of women in maternal healthcare settings has become a public health concern, contributing to a significant barrier to reducing maternal morbidity and mortality (Habib et al. 2023; Mannava et al. 2015). Obstetric mistreatment, which refers to abusive and harmful practices occurring during pregnancy, childbirth, and the postpartum period, is a violation of the rights and dignity of women (Chervenak et al. 2024). Studies have documented diverse experiences of abuse and disrespect among women during maternal care (Dzomeku et al. 2020; Ganle and Krampah 2019; Malatji and Madiba 2020; Mapumulo et al. 2021; Oluoch‐Aridi et al. 2018; Orpin et al. 2018). Systematic reviews across diverse regions have reported that women of reproductive age are more likely to experience disrespectful maternal care, including physical abuse, verbal abuse, stigma, discrimination, neglect, abandonment, forced sterilization, and breaches of confidentiality and consent (Bohren et al. 2015; Fraser et al. 2025; Gebeyehu et al. 2023; Kassa et al. 2020; Mengesha et al. 2020). Evidence shows that about 20% of women in Europe (Lukasse et al. 2015), 20% in the United States (US) (CDC 2023), 43% in Latin America (Tobasía‐Hege et al. 2019), and 44% in Sub‐Saharan Africa (Kassa et al. 2020), have experienced some form of obstetric mistreatment. Furthermore, studies have also shown that obstetric mistreatment may be even more prevalent among women living with HIV, mostly due to HIV‐related stigma, discrimination, and abuse within healthcare settings, which may be more pronounced and exacerbated (Jan et al. 2023; Subramaniyan et al. 2013; Yigit et al. 2025).

Although some studies have investigated whether women living with HIV are at greater risk of experiencing mistreatment than HIV negative women in healthcare settings, there are mixed findings depending on the type of mistreatment (Sando et al. 2014; Appiah et al. 2023; Sethi et al. 2017). For example, Sando et al. reported a similar prevalence of disrespect and abuse between HIV‐negative women (15%) and those living with HIV (12.20%) (Sando et al. 2014). Similarly, Appiah et al. observed a similar prevalence of any form of obstetric mistreatment between women living with HIV (61%) and HIV‐negative women (65.1%) (Appiah et al. 2023). However, some studies have shown that women living with HIV significantly experience more stigma and discrimination, and a lack of informed consent, than HIV‐negative women (Sando et al. 2014; Cuca and Rose 2016; Colombini et al. 2014; Ashaba et al. 2017). For instance, Sando et al. observed in their study that 100% of women living with HIV experienced non‐consented care (e.g., vaginal examinations without consent during labor) compared to 80% of HIV‐negative women (Sando et al. 2014).

Despite the mixed results in the literature, a report by the International Community of women (ICW) in 2024 entitled “Confronting Coercion” calls attention to the ongoing experiences of violation encountered by women living with HIV when accessing sexual and reproductive health services (International Community of Women Living with HIV 2024). The ICW has observed persistent exposure of women living with HIV to coercive practices, including forced sterilization and mistreatment in healthcare settings (International Community of Women Living with HIV 2024). Using the Stigma Index 2.0 to explore disrespectful maternal care practices among 26 502 women living with HIV from over 23 countries between 2020 and 2023, ICW findings revealed that about 20% of women living with HIV have been coerced at some point in their lifetime related to issues with pregnancy, childbirth, sterilization, contraception, and family planning (International Community of Women Living with HIV 2024). In addition, 4.4% of women living with HIV reported being coerced over the past 12 months, and 4% reported experiencing one form of abuse and mistreatment in the past 12 months, and this varies by region (International Community of Women Living with HIV 2024). These data suggest that women living with HIV are disproportionately affected and highlight the urgent need for sustainable interventions in improving health outcomes.

While most systematic reviews have focused mainly on the experiences of mistreatment among women in general in maternal healthcare settings (Gebeyehu et al. 2023; Mohamoud 2023; Bohren et al. 2019; Vedam et al. 2019; Maung et al. 2021; Kasaye et al. 2024; Sudhinaraset et al. 2016), no study has examined in detail the experiences of women living with HIV when accessing maternal care. This systematic review, using the advanced convergent meta‐integration framework, aims to synthesize the existing quantitative and qualitative literature and comprehensively assess the prevalence of and factors that influence obstetric mistreatment among women living with HIV.

### 1.1 Theoretical Framework

We employed the definition of obstetric mistreatment as described by Bohren et al. and Chervenak et al., which refers to a wide range of disrespectful and abusive practices, whether intentional or unintentional, occurring during pregnancy, delivery, and the post‐partum period in maternal healthcare settings (Chervenak et al. 2024; Bohren et al. 2015). We derived our themes from the “typology of mistreatment,” based on global evidence, as described by Bohren et al. (2015). Bohren et al. developed a typology of mistreatment among women during childbirth based on evidence from 65 studies across 34 countries, organized into three levels: first‐order, second‐order, and third‐order themes. The third‐order themes comprise seven broad categories of mistreatment, encompassing both first‐ and second‐level themes. The third‐order themes are: (1) physical abuse, (2) sexual abuse, (3) verbal abuse, (4) stigma and discrimination, (5) failure to meet professional standards of care, (6) poor rapport between women and providers, (7) health system conditions and constraints.

The second‐order themes are subcategories within each third‐order theme. For instance; physical abuse (e.g., use of force, physical restraint), sexual abuse (e.g., sexual abuse), verbal abuse (e.g., harsh language, threats, and blaming), stigma and discrimination (e.g., discrimination based on sociodemographic characteristics and medical conditions), failure to meet professional standards of care (e.g., lack of informed consent and confidentiality, physical examinations and procedures, neglect and abandonment), poor rapport between women and providers (e.g., ineffective communication, lack of supportive care, and loss of autonomy), health systems and conditions and constraints (e.g., lack of resources, lack of policies, and facility culture).

The first‐order themes were synthesized directly from participants' quotes reported in the included studies (Bohren et al. 2015). For example, within the domain of physical abuse, first‐order themes included women living with HIV's reports of being beaten, pinched, slapped, or physically restrained to a bed (Bohren et al. 2015). From the foregoing, this review adopts the typology of mistreatment to categorize and document different forms of abuse or mistreatment experienced by women living with HIV in health facilities.

## 2 Methods

This review followed the advanced convergent meta‐integration framework proposed by Frantzen and Fetters to guide the synthesis of qualitative and quantitative findings (Frantzen and Fetters 2016). This review was reported in accordance with the Updated Preferred Reporting Items for Systematic Review and Meta‐Analysis (PRISMA) guidelines (Page et al. 2021). Appendix S1 outlines the PRISMA checklist for this review. This review was registered on PROSPERO (ID: CRD42024498615).

### 2.1 Eligibility Criteria

The eligibility criteria were adapted using the Population, Exposure, and Outcome (PECO) framework (Moola et al. 2015). Population: We included studies on women in all age groups. Exposure: Maternal delivery in a community‐based or healthcare facility. Comparator: Studies with or without a comparator group were considered. Outcome: We evaluated the prevalence and associated factors of obstetric mistreatment. Study design: We considered quantitative studies (prospective cohort, retrospective cohort, case–control, and cross‐sectional), qualitative studies, and mixed‐method studies with evidence of obstetric mistreatment among women living with HIV. We did not restrict our search by date or language. We excluded commentary articles, reviews, conference abstracts, editorials, letters, case reports, and protocols.

### 2.2 Database and Search Strategy

Two reviewers (JE and OB) conducted systematic searches across five databases—PubMed, Embase, CINAHL, SCOPUS, and Web of Science. In addition, we searched Google Scholar to complement the primary databases and identify more relevant papers, as it indexes a broad range of sources that may not be captured by them. Moreover, Google Scholar has been found useful for identifying relevant papers for reviews (Mahood et al. 2014). We also hand‐searched the reference lists and citations of included studies. We searched the databases in January 2024 and updated the search in January 2025. The search strategy combined two searches, one related to women living with HIV and one related to obstetric mistreatment, using the following search terms: women living with HIV, WLWH, WLHIV, WLWHA, HIV positive women, HIV infected women, obstetric violence, obstetric mistreatment, mistreatment, disrespect and abuse, disrespect, humiliation and verbal abuse, verbal abuse, physical abuse, non‐consented care, non‐confidential care, lack of privacy, undignified care, abandonment, abandonment during labor, abandonment during delivery, abandonment of care, abandonment after labor, abandonment after delivery, detention in facility, discriminated care, non‐dignified care, forced sterilization, coerced sterilization, unconsented medical procedure, violation of privacy, refusal of admission, physical violence, neglected care, microaggression, negative birth experiences, stigma and discrimination, stigma, discrimination. As an example, we used the following keywords to identify relevant studies in PubMed: (“women living with HIV” OR WLHIV OR WLWH OR WLWHA OR “HIV positive women” OR “HIV infected women”) AND (“obstetric violence” OR “obstetric mistreatment” OR mistreatment OR “disrespect and abuse” OR disrespect OR “humiliation and verbal abuse” OR “verbal abuse” OR “physical abuse” OR “non‐consented care” OR “non‐confidential care” OR “lack of privacy” OR “undignified care” OR abandonment OR “abandonment during labor” OR “abandonment during delivery” OR “abandonment of care” OR “abandonment after labor” OR “abandonment after delivery” OR “detention in facilit*” OR “discriminated care” OR “non‐dignified care” OR “forced sterilization” OR “coerced sterilization” OR “unconsented medical procedure*” OR “violation of privacy” OR “refusal of admission” OR “physical violence” OR “neglected care” OR “microaggression” OR “negative birth experiences” OR “stigma and discrimination” OR stigma OR discrimination). Appendix S2 contains the full search strategy for all the databases.

### 2.3 Study Selection

Two reviewers (JE and OB) conducted the systematic search, and the results were compiled and exported to the Rayyan systematic review manager. One reviewer (JE) conducted deduplication. JE and OB independently screened the titles and abstracts, and the full texts were reviewed to determine whether they met the eligibility criteria. Our screening percentage agreement was 91.2% between the two reviewers. Articles on which the independent reviewers disagreed were subsequently re‐evaluated through discussions until a consensus was reached. Therefore, there was no need to invite a third reviewer to settle the disagreement.

### 2.4 Data Extraction

Two reviewers (JE and RA) independently extracted information from the included studies, which was then verified separately by OB. We adapted an abstraction sheet from previous studies (Adeagbo et al. 2025; Badru, Edeh, et al. 2025; Badru, Alabi, et al. 2025), and extracted the following information: author and year of publication, study setting (community or health facility), study design (observational: quantitative or qualitative), sample size, characteristics of the participants (such as mean or median or modal age), data collection methods, study sampling, forms of obstetric mistreatment (frequency and percentages), and data analysis. We extracted relevant verbatim quotations from the qualitative studies. Appendix S3 provides representative quotes from the included studies.

### 2.5 Quality Assessment

We appraised the quality of the qualitative studies using the appropriate Critical Appraisal Skills Programme (CASP) tool (CASP Qualitative Studies Checklist 2023). The CASP checklist tools comprised 10 criteria for qualitative studies, covering the study's objectives, methodology, research design, recruitment strategy, data collection, data analysis, the researcher's influence on the study, clarity of research findings, ethical considerations, and the research contribution. Each CASP item was scored as follows: 2 = criterion fully met, 1 = partially met, and 0 = not met. We adapted the CASP checklist rating from a previous study (Njau et al. 2019). A score of 20 indicates high quality, 16–19 indicates moderate quality, and ≤ 15 indicates low quality (Njau et al. 2019). Appendix S4 shows all items on the CASP qualitative appraisal tool and the score for each study.

The quality assessment for the cross‐sectional studies was conducted using the Joanna Briggs Institute (JBI) Critical Appraisal Checklist. The JBI checklist comprises eight criteria, with one point awarded for each criterion met. If the criterion was fully satisfied, it was given 1 point; 0 points if it was not satisfied or was unclear. We adapted JBI scoring according to a recent study (Fei et al. 2025). A score of 0–3 is considered low quality, 4–6 is moderate quality, and 7–8 is high quality (Fei et al. 2025).

For mixed‐method studies, we utilized the Mixed Methods Appraisal Tool (MMAT) version 2018 (Hong et al. 2018), and rated each study based on the mixed‐method components. The criteria for each category were rated “Yes,” meaning the criteria were fulfilled, “No,” which means the criteria were not met, and “Can't tell,” meaning there is insufficient information to assess whether the criteria were met or not. We adapted the MMAT rating from a previous study (Astawesegn et al. 2024). Studies were categorized as high, moderate, or low based on whether they met the criteria, with a total score of 7. Studies that scored ≥ 6 were considered high quality, studies that scored 3–5 were classified as moderate quality, and those with ≤ 2 were low quality (Astawesegn et al. 2024). Three reviewers (JE, RA, and EA) independently appraised the studies and met to discuss discrepancies.

### 2.6 Data Synthesis

As our review included a heterogeneous sample of studies, we employed a convergent mixed‐method systematic review approach to enable the concurrent synthesis and integration of quantitative, qualitative, and mixed‐method studies (Frantzen and Fetters 2016). The convergent synthesis design is more appropriate for this review, as the same phenomenon of interest was addressed using both quantitative and qualitative methods. We utilized the advanced convergent meta‐integration model without transformation proposed by Frantzen and Fetters, which is designed to accommodate findings from qualitative, quantitative, and mixed‐method studies. Therefore, quantitative values (e.g., prevalence rates or effect sizes) were retained to preserve important contextual information, particularly when precise numbers were critical for understanding the magnitude or frequency of the phenomena under study. Mixed‐method studies were fractionated, that is, their quantitative and qualitative components were separated into separate datasets (Frantzen and Fetters 2016). In circumstances where either quantitative or qualitative data were available and could not be integrated, findings were reported separately. The synthesized quantitative and qualitative data demonstrating similar patterns were reported in accordance with Bohren's theoretical framework (i.e., deductive approach), using the third‐order themes (Bohren et al. 2015). To reduce redundancy, we merged overlapping themes observed in our study.

Notably, we did not pool prevalence estimates and associated factors for mistreatment due to substantial variation in the measured outcomes across studies. Moreover, some factors were reported in a single study, making pooling of these factors impossible. Hence, a descriptive comparison of both quantitative and qualitative data reporting the same outcome was performed to assess similar and divergent findings.

## 3 Results

**FIGURE 1:**
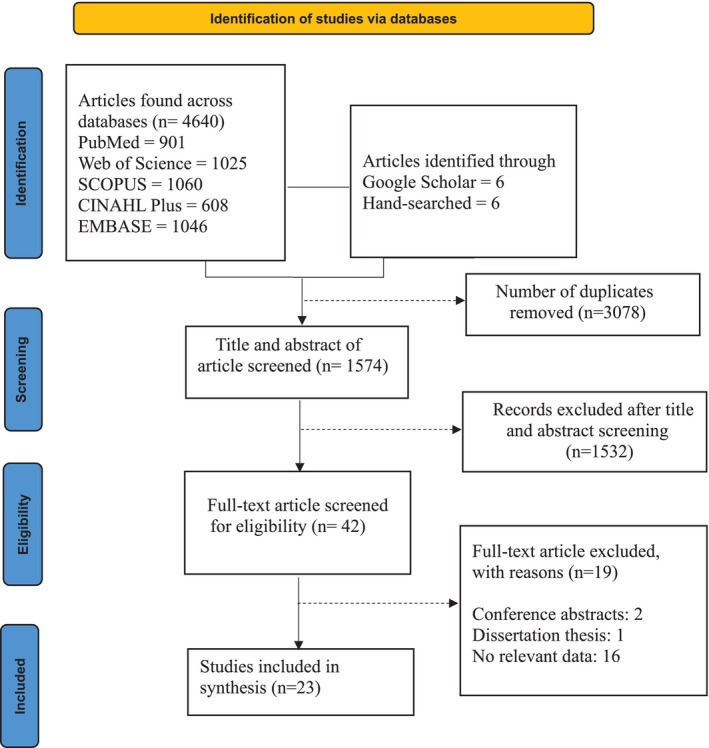
Search strategy flowchart for obstetric mistreatment among women with HIV.

We identified 4652 articles across the six databases for potential inclusion. Of these, 3078 duplicates were removed, and 1574 were subjected to title and abstract screening. We excluded 1532 studies that did not meet the inclusion criteria, leaving 42 for full‐text screening. Nineteen articles after full‐text screening were excluded for the following reasons: not relevant to obstetric mistreatment (n: 16), conference abstracts (n: 2), and dissertation thesis (n: 1). Twenty‐three articles were deemed eligible and included in this study (Figure 1).

### 3.1 Characteristics of Included Studies

Of the 23 studies included in this review, 15 were qualitative (Jan et al. 2023; Cuca and Rose 2016; Ashaba et al. 2017; Greene et al. 2016; Arrey et al. 2017; Bakare and Gentz 2020; Weber et al. 2024; Strode et al. 2012; Gourlay et al. 2014; Onono et al. 2015; Kelly et al. 2013; Valencia‐Garcia et al. 2017; Malta et al. 2010; Nguyen et al. 2024; Madhivanan et al. 2014), three were quantitative (Appiah et al. 2023; Sethi et al. 2017; Assefa et al. 2022), and five were mixed‐method studies (Sando et al. 2014; Kendall and Albert 2015; Lester et al. 1995; Women of the Asia Pacific Network of People Living with HIV 2012; Barabara et al. 2025). Three studies were conducted in Tanzania (Sando et al. 2014; Gourlay et al. 2014; Barabara et al. 2025), two each in South Africa (Weber et al. 2024; Strode et al. 2012), the US (Cuca and Rose 2016; Lester et al. 1995), and India (Jan et al. 2023; Madhivanan et al. 2014). One study was conducted in Ghana (Appiah et al. 2023), Uganda (Ashaba et al. 2017), Ethiopia (Assefa et al. 2022), Canada (Greene et al. 2016), Belgium (Arrey et al. 2017), Namibia (Bakare and Gentz 2020), Kenya (Onono et al. 2015), Malawi (Sethi et al. 2017), Peru (Valencia‐Garcia et al. 2017), Brazil (Malta et al. 2010), Vietnam (Nguyen et al. 2024), and Northern Ireland (Kelly et al. 2013). One study focused on four Latin American countries: El Salvador, Honduras, Mexico, and Nicaragua (Kendall and Albert 2015). One study was conducted in six Asian countries: Bangladesh, Cambodia, India, Indonesia, Nepal, and Vietnam (Women of the Asia Pacific Network of People Living with HIV 2012). Using the World Health Organization (WHO) regional classification (Table 1), 11 studies were conducted in the African region (Sando et al. 2014; Appiah et al. 2023; Sethi et al. 2017; Ashaba et al. 2017; Bakare and Gentz 2020; Weber et al. 2024; Strode et al. 2012; Gourlay et al. 2014; Onono et al. 2015; Assefa et al. 2022; Barabara et al. 2025), five in the Americas region (Greene et al. 2016; Valencia‐Garcia et al. 2017; Malta et al. 2010; Kendall and Albert 2015; Lester et al. 1995), two in the European region (Arrey et al. 2017; Kelly et al. 2013), two in the Southeast Asia region (Jan et al. 2023; Madhivanan et al. 2014), and one in the Western Pacific region (Nguyen et al. 2024). One study was conducted in both Southeast Asia and the Western Pacific regions (Women of the Asia Pacific Network of People Living with HIV 2012).

**TABLE 1:** Characteristics of the included studies.

Author	Country/Setting	Study design	Sample size	Study sampling	Obstetric mistreatment types	Data collection methods	Participant's characteristics	Obstetric mistreatment type *N* (%)	Evidence level
Sando et al. (2014)	Tanzania/Hospital‐based	Mixed Methods	147	Purposive sampling	Any form of disrespect or abuse Physical abuse Non‐dignified care Non‐consented care Non‐confidential care Lack of privacy Abandonment Detention in a facility	In‐depth interview Direct observations Structured questionnaire	Mean age (standard deviation): 29.1 ± 8.1	Any form of disrespect or abuse: 18 (12.2) Physical abuse: 4 (2.7) Non‐dignified care: 7 (4.8) Non‐consented care: 2 (1.4) Non‐confidential care: 1 (0.7) Lack of privacy: 1 (0.7) Abandonment: 10 (6.8) Detention: 1 (0.7) **Direct clinical observations** **Physical abuse** Episiotomy administered without anesthesia: 1(5.6) **Non‐consented care** Non‐consented first examination: 16 (89) Non‐consented vaginal examination: 18 (100) **Non‐confidential care** Medical history discussed in the presence of others: 4 (22) **Lack of privacy** Partitions did not effectively provide privacy: 17 (94.4) Body not adequately covered during labor and delivery: 12 (66.7) **Non‐dignified care** Verbal abuse: 1 (5.6) Provider does not call woman by her name throughout the duration of the visit: 6(35.3) The provider did not offer congratulatory remarks following delivery: (47.1) Not cleaned up following delivery: 3 (17.7) Lack of clean bed in the postnatal ward: 12 (70.6)	High quality
Appiah et al. (2023)	Ghana/Hospital‐based	Cross‐sectional study	310	Purposive sampling	Any form of Obstetric violence Non‐dignified care Non‐consented care Discriminated care Non‐confidential care Neglected care or abandoned care. Detention Physical abuse	Questionnaire	Modal age group: > 30 years: 198 (63.9)	Any form of Obstetric violence: 189 (61.0) Non‐dignified care: 66 (21.3) Non‐consented care: 11 (3.5) Discriminated care: 42 (13.5) Non‐confidential care: 103 (32.3) Neglected care or abandoned care: 101 (32.6) Detention: 21 (6.8) Physical abuse: 48 (15.5)	High quality
Assefa et al. (2022)	Ethiopia/Hospital‐based	Cross‐sectional study	318	Systematic random sampling	Failure to get fully informed consent. Physical violence Unconsented medical procedures. Gross violation of privacy and confidentiality Profound humiliation and verbal abuse Neglecting to suffer life‐threatening complications Refusal of admission to the health care facility	Questionnaires	Mean age: 29.45 ± 6.28	Overall prevalence: (79%) Failure to get fully informed consent: 249 (78.3) Physical violence: 104 (32.7) Unconsented medical procedures: 227 (71.4) Gross violation of privacy and confidentiality: 137 (43) Profound humiliation and verbal abuse: 99(31) Neglecting to suffer life‐threatening complications: 140 (44) Refusal of admission to the health care facility: 49 (15.5)	High quality
Greene et al. (2016)	Canada/Community‐based/Obstetrical health care centers	Qualitative	66 pregnant women (Third trimester) 64 women (early postpartum)	—	HIV‐related stigma	Face‐to‐face interviews	Median age: 33 years Age range: 21–42 years	—	Moderate quality
Kendall and Albert (2015)	Four Latin American countries (EL Salvador, Honduras, Mexico, and Nicaragua)/Community‐based.	Mixed Method	285	Convenience sampling	Forced and coerced sterilization	Questionnaires based on international human rights law	Modal age group: 25–34: 100 (36%)	Pressured to sterilize post‐diagnosis; Nicaragua: 17% Honduras: 22% El Salvador: 23% Mexico: 28% Total: 23%	High quality
Arrey et al. (2017)	Belgium/Community‐based (AIDS Reference Centre, support group sites, and HIV workshop venues)	Qualitative	44	Purposive and snowball samplings	HIV‐related stigma and discrimination Non‐confidential care	In‐depth interviews Semi‐structured interviews Observations	Modal age group: 40–49: 15 (34.1%)	—	Moderate quality
Bakare and Gentz (2020)	Namibia/Community‐based (NGO Foundation)	Qualitative	7	Convenience sampling	Forced sterilization	Face‐to‐face interviews	Age range: 38–44	—	Moderate quality
Lester et al. (1995)	United States/Hospital‐based	Mixed method	20	Purposive sampling	HIV‐related stigma Discrimination Lack of informed consent	Interview	Age range: 20–42	Discrimination: 7 (35%)	High quality
Jan et al. (2023)	Indian/Hospital‐based	Qualitative	21	Purposive sampling	Stigma Discrimination Verbal abuse Denial of care and treatment Non‐confidential care	Semi‐structured Interview	Age range: 20–70	—	Low quality
Weber et al. (2024)	South Africa/Community‐based settings (community healthcare clinic)	Qualitative	26 (Postpartum women at 6–8 weeks)	Purposive sampling	Lack of informed consent Disrespect Inequitable care Harm and Ill‐treatment Denied access to care Non‐consensual child separation Negligence Inadequate information	In‐depth Interviews	Mean age: 28 ± 6.4	—	Moderate quality
Strode et al. (2012)	South Africa/Community‐based	Qualitative	22	Purposive and snowball samplings	Forced sterilization without knowledge and consent	Semi‐structured interview	—	Sterilized without consent: 4 (18.2%) Involuntary sterilization: 18 (81.8%)	Moderate Quality
Gourlay et al. (2014)	Tanzania/Hospital‐based	Qualitative	16	Random and purposive sampling	Discrimination Stigma	In‐depth interviews Observations	Mean age: 36 Age range: 19–54	—	Moderate Quality
Barabara et al. (2025)	Tanzania/Hospital‐based and community‐based settings	Mixed method	103	Purposive sampling	Stigma Discrimination Verbal abuse Neglect Lack of confidentiality Lack of supportive care Lack of privacy Lack of consent Failure to provide adequate information	Surveys In‐depth interviews	Median Age: 28 (IQR: 26–34)	Physical abuse: 15 (15%) Verbal abuse: 21 (20.4%) Lack of visual privacy during labor: 30 (29%) Prolonged period of waiting for care: 34 (33%) Denied family/friend support during labor or delivery: 29 (28%) Not consented before performing a procedure: 17 (16.5%) Denied their position of choice during delivery: 18 (17.5%) Not included in their care: 4 (3.9%) Inadequate information on the procedures conducted: 15 (14.6%) Providers did not help to control pain: 16 (15.5%) Participants were unable to talk about their feelings with providers: 16 (15.5%) Inadequate staff: 12 (11.7%) Lack of clean facilities: 11 (10.7) Providers did not provide support for anxiety and fears: 9 (8.7)	High Quality
								Unable to ask questions about their health: 10 (9.7%) Medical records were not kept confidential: 6 (5.8%) Provider did not provide the best care: 5 (4.9%) Participants did not provide help when needed: 4 (3.9) Lack of water in the facility: 4 (3.9%) Lack of trust in providers in the facility: 3 (2.9%) Participants did not understand provider's language: 2 (1.9%) Disrespect: 2 (1.3%) Lack of friendly providers: 1 (1.0%) Lack of electricity in the facility: 2 (1.9%) Lack of safe facility: 1 (1.0%)	
Onono et al. (2015)	Kenya/Community‐based	Qualitative	33	Purposive sampling	Stigma Discrimination Ill‐treatment Neglect Denial of adequate information	In‐depth interviews	Mean age: 27.1 ± 5.0	—	Moderate Quality
Kelly et al. (2013)	Northern Ireland/Hospital‐based	Qualitative	15	Purposive sampling	Breeches of confidentiality Conflicted medical advice Discrimination Inappropriate care Lack of consent	In‐depth interviews	Age range: 20–40	—	Moderate Quality
Sethi et al. (2017)	Malawi/Hospital‐based	Quantitative	137	Convenience sampling	Non‐dignified care Non‐consented care Non‐confidential care Abandonment or denial of care	Direct clinical observations	Median age: 23	Obstetric mistreatment experienced by women compared to those not living with HIV **Non‐dignified care:** Provider did not respectfully greet women: OR: 1.21, *p* = 0.53 Provider shouted, insulted or threatened the woman during labor or after: OR: 1.46, *p* = 0.53 **Non‐consented care** The provider did not give at least one update on the progress of labor: OR: 1.26, *p* = 0.43 The provider did not ask if patients had any questions: OR: 0.81, *p* = 0.68 The provider did not ask if patients have any other problems they were concerned about: OR: 0.08, *p* = 0.04 The provider did not explain procedures to patients before conducting them: OR: 0.62, *p* = 0.56 The provider did not inform the patients what will occur before the vaginal examination: OR: 0.74, *p* = 0.64 No information provided on findings: OR: 0.46, *p* = 0.46 No explanation on what will happen in the labor room at least once: OR: 1.05, *p* = 0.93 **Lack of privacy** No provision of audio and visual privacy: OR: 0.34, *p* = 0.05 No provision of drape: OR: 1.13, *p* = 0.84 Provider does not use visual coverings to cover woman during examinations, and procedures: OR: 0.38, *p* = 0.23. **Supportive care** Provider did not encourage patients to have a support person during delivery: OR: 0.64, *p* = 0.50	Moderate quality
								**Abandonment or denial of care** Provider did not encourage participants to take food/fluids at least once: OR: 1.59; *p* = 0.40 Provider did not encourage participants to ambulate at least once during labor: OR: 0.65, *p* = 0.54	
Ashaba et al. (2017)	Uganda/Hospital‐based	Qualitative	20	Purposive sampling	Stigma Discrimination Lack of supportive care	One‐on‐one in‐depth interviews	Median age: 33 (IQR: 28–35)	—	Moderate quality
Cuca and Rose (2016)	United States/Community‐based	Qualitative	20	Purposive sampling	Stigma	In‐depth interview	Mean age: 46 Age range: 35–60	—	Moderate quality
Valencia‐Garcia et al. (2017)	Peru/Community‐based	Qualitative	14	Purposive	Stigma Discrimination Non‐confidential care Lack of privacy	Face‐to‐face interviews	Mean age: 33 Age range: 20–47	—	Moderate quality
Malta et al. (2010)	Brazil/Community‐based	Qualitative	70	Purposive	Stigma Forced sterilization Lack of support from providers	Focus‐group discussions In‐depth interviews Freelist interviews	Mean age: 28.3 Age range: 18–40	—	Moderate quality
Nguyen et al. (2024)	Vietnam/Community‐based	Qualitative	30	Snowballing	HIV stigma and Discrimination Forced sterilization Lack of informed consent Lack of appropriate care Lack of adequate information	Semi‐structured interviews	Modal age: 36–45: 15 (50.0%)	—	Moderate quality
Madhivanan et al. (2014)	India/Community‐based/(women support groups, Government of India Integrated Counseling and Testing Centers, and non‐governmental organizations)	Qualitative	14	Purposive	Lack of confidentiality Lack of informed consent Stigma Discrimination	In‐depth interviews	Mean age: 23	—	Moderate quality
Women of the Asia Pacific Network of People Living with HIV (2012)	Six Asian countries: Bangladesh, Cambodia, India, Indonesia, Nepal, and Vietnam	Mixed Method	757	—	Stigma Discrimination Forced sterilization Neglect Lack of supportive care from healthcare providers Lack of confidentiality Denied access to care	Questionnaire In‐depth interview Focus group discussions	Mean age: 29.3 Age range: 17–47	Unable to decline forced sterilization (37.7%)	High quality

Abbreviations: IQR: interquartile range; OR: odds ratio.

Across the 23 studies, approximately 2500 women living with HIV were recruited from various settings, including public and private hospitals, NGOs, and HIV awareness workshop sites. Most studies recruited participants who had given birth in public or private hospitals and not at home (Jan et al. 2023; Sando et al. 2014; Appiah et al. 2023; Sethi et al. 2017; Ashaba et al. 2017; Gourlay et al. 2014; Kelly et al. 2013; Madhivanan et al. 2014; Assefa et al. 2022; Lester et al. 1995). Participants were also recruited from community‐based settings, including women's support groups, NGOs, and HIV awareness workshop sites (Cuca and Rose 2016; Greene et al. 2016; Arrey et al. 2017; Bakare and Gentz 2020; Weber et al. 2024; Strode et al. 2012; Onono et al. 2015; Valencia‐Garcia et al. 2017; Malta et al. 2010; Nguyen et al. 2024; Madhivanan et al. 2014; Kendall and Albert 2015). One study included women living with HIV who had delivered in either community‐ or hospital‐based settings (Barabara et al. 2025). In addition, while most studies utilized self‐reports of mistreatment from women living with HIV (Jan et al. 2023; Appiah et al. 2023; Cuca and Rose 2016; Ashaba et al. 2017; Greene et al. 2016; Arrey et al. 2017; Bakare and Gentz 2020; Weber et al. 2024; Strode et al. 2012; Gourlay et al. 2014; Onono et al. 2015; Kelly et al. 2013; Valencia‐Garcia et al. 2017; Malta et al. 2010; Nguyen et al. 2024; Madhivanan et al. 2014; Assefa et al. 2022; Kendall and Albert 2015; Lester et al. 1995; Barabara et al. 2025), three studies relied on both self‐reports and direct observations during labor and childbirth (Sando et al. 2014; Arrey et al. 2017; Gourlay et al. 2014). One study was based solely on direct clinical observations (Sethi et al. 2017).

There was variability in the type of obstetric mistreatment measured and reported. Measurement tools for obstetric mistreatment included Bowser and Hill's, Bohren's evidence‐based typology of mistreatment, and the Person‐Centered Maternity Care (PCMC). Most studies reported HIV‐related stigma/discrimination (Jan et al. 2023; Appiah et al. 2023; Cuca and Rose 2016; Ashaba et al. 2017; Greene et al. 2016; Arrey et al. 2017; Gourlay et al. 2014; Onono et al. 2015; Kelly et al. 2013; Valencia‐Garcia et al. 2017; Malta et al. 2010; Nguyen et al. 2024; Madhivanan et al. 2014; Lester et al. 1995; Lester et al. 1995; Women of the Asia Pacific Network of People Living with HIV 2012; Barabara et al. 2025). Twelve studies reported abandonment/neglect, or lack of supportive care, or denial of care (Jan et al. 2023; Sando et al. 2014; Appiah et al. 2023; Sethi et al. 2017; Arrey et al. 2017; Weber et al. 2024; Onono et al. 2015; Valencia‐Garcia et al. 2017; Malta et al. 2010; Assefa et al. 2022; Women of the Asia Pacific Network of People Living with HIV 2012; Barabara et al. 2025). Eleven studies were on non‐confidential care (Jan et al. 2023; Sando et al. 2014; Appiah et al. 2023; Sethi et al. 2017; Arrey et al. 2017; Kelly et al. 2013; Valencia‐Garcia et al. 2017; Madhivanan et al. 2014; Assefa et al. 2022; Lester et al. 1995; Women of the Asia Pacific Network of People Living with HIV 2012), nine on lack of consent (Sando et al. 2014; Appiah et al. 2023; Sethi et al. 2017; Weber et al. 2024; Kelly et al. 2013; Nguyen et al. 2024; Madhivanan et al. 2014; Assefa et al. 2022; Barabara et al. 2025), nine on non‐dignified care (including verbal abuse) (Jan et al. 2023; Sando et al. 2014; Appiah et al. 2023; Sethi et al. 2017; Gourlay et al. 2014; Onono et al. 2015; Assefa et al. 2022; Barabara et al. 2025), seven on failure to provide adequate information (Weber et al. 2024; Strode et al. 2012; Onono et al. 2015; Kelly et al. 2013; Valencia‐Garcia et al. 2017; Nguyen et al. 2024; Barabara et al. 2025), six on forced/coerced sterilization (Bakare and Gentz 2020; Strode et al. 2012; Malta et al. 2010; Nguyen et al. 2024; Kendall and Albert 2015; Women of the Asia Pacific Network of People Living with HIV 2012), four on lack of privacy (Sando et al. 2014; Valencia‐Garcia et al. 2017; Assefa et al. 2022; Barabara et al. 2025), four on physical abuse (Sando et al. 2014; Appiah et al. 2023; Assefa et al. 2022; Barabara et al. 2025), three on extortion and bribery (Gourlay et al. 2014; Nguyen et al. 2024; Women of the Asia Pacific Network of People Living with HIV 2012), two on detention in healthcare facilities (Sando et al. 2014; Appiah et al. 2023). One study was on non‐consensual child separation (Weber et al. 2024).

The sampling methods utilized across the studies also varied. Most studies used purposive sampling (Jan et al. 2023; Sando et al. 2014; Appiah et al. 2023; Cuca and Rose 2016; Ashaba et al. 2017; Weber et al. 2024; Onono et al. 2015; Kelly et al. 2013; Valencia‐Garcia et al. 2017; Malta et al. 2010; Madhivanan et al. 2014; Lester et al. 1995; Barabara et al. 2025). Three studies utilized convenience sampling (Sethi et al. 2017; Bakare and Gentz 2020; Kendall and Albert 2015), and two utilized purposive and snowball sampling (Arrey et al. 2017; Strode et al. 2012). One study each was on purposive and random sampling techniques (Gourlay et al. 2014), systematic random technique (Assefa et al. 2022), and snowballing (Nguyen et al. 2024). However, two studies did not report their sampling technique (Greene et al. 2016; Women of the Asia Pacific Network of People Living with HIV 2012).

### 3.2 Risk of Bias Assessment

Regarding the three cross‐sectional studies assessed using JBI, two studies were considered high quality with a score of eight (Appiah et al. 2023; Assefa et al. 2022), and one was of moderate quality, scoring six because they did not adjust for confounders (Sethi et al. 2017). Fourteen of the 15 qualitative studies were of moderate quality, scoring 17 or 18. One study received a lower score of 11 due to insufficient detail on the methodology, including participant recruitment, data collection methods, and data analysis methods (Jan et al. 2023). All the qualitative studies included also failed to address the researcher's positionality and how the relationship between researchers might have influenced their findings. For the mixed methods studies, three received a total quality appraisal score of seven, while two studies scored six; all were considered high quality.

### 3.3 Any Form of Obstetric Mistreatment

The description of obstetric mistreatment across the studies is described below. We identified various sub‐themes within the categories of the third‐order typology by Bohren et al. (2015). Three quantitative studies reported the prevalence rate of any form of obstetric mistreatment (Sando et al. 2014; Appiah et al. 2023; Assefa et al. 2022). We observed an overall prevalence of any form of obstetric mistreatment ranging from 12.2% to 79%, with the lowest reported in Tanzania (12.2%), followed by Ghana (61%), and the highest in Ethiopia (79%) (Sando et al. 2014; Appiah et al. 2023; Assefa et al. 2022). Several factors, such as age, education, marital status, being delivered by a female attendant, and frequency of antenatal visits, have been associated with the mistreatment of women living with HIV in maternal care settings (Appiah et al. 2023; Assefa et al. 2022; Kendall and Albert 2015). For instance, Assefa et al. reported that women living with HIV in Ethiopia aged 35 and above were twice as likely to experience obstetric mistreatment as their younger counterparts between 15 and 24 (adjusted odds ratio [AOR]: 2.47, 95% CI: 1.25–4.90) (Assefa et al. 2022). Women living with HIV who had received care from female birth attendants were about threefold more likely to be mistreated compared to those delivered by male birth attendants (AOR: 2.85, 95% CI: 1.58–5.15) (Assefa et al. 2022). Additionally, women living with HIV who had three antenatal care visits experienced mistreatment from healthcare providers (AOR: 2.99, 95% CI: 1.39–6.45) (Assefa et al. 2022). The educational status of women living with HIV also played a role in determining whether they experienced maltreatment in maternal healthcare settings. Women living with HIV in Ethiopia with a primary education had twice the odds (AOR: 2.13, 95% CI: 1.08–4.17) of being mistreated compared to those with secondary or higher educational levels (Assefa et al. 2022). However, the study observed no significant association between occupational status (government or private employee vs. housewife), number of birth attendants (1–3 or 4–6 vs. > 6), or time of delivery (day versus night) and obstetric mistreatment among women living with HIV (Assefa et al. 2022).

Appiah et al. reported that marital status was a significant predictor of any form of obstetric mistreatment among women (those living with and not living with HIV) (Appiah et al. 2023). Single women in Ghana were 52% more likely to experience any form of obstetric mistreatment compared to married, divorced, widowed, and those living with a partner (AOR: 1.52, 95% CI: 1.15–2.02) (Appiah et al. 2023). Teenage mothers (15–19 years) in Ghana were found to have increased odds of being physically abused compared to older adults (AOR: 2.5, 95% CI: 1.45–4.17) (Appiah et al. 2023). The study also reported that women in Ghana with no formal education were more predisposed to experiencing discrimination (AOR: 1.56, CI: 1.01–2.42) than those with some level of education (Appiah et al. 2023). Women with no formal education were more likely to experience non‐consented care (AOR: 1.73, 95% CI: 1.02–2.92) than their educated counterparts (Appiah et al. 2023). Women in the 20–29 age range had lower odds of being neglected following delivery compared to those above 30 years of age (AOR: 0.80, 95% CI: 0.66–0.98) (Appiah et al. 2023). However, the study did not report whether this association differed specifically for women with HIV compared to HIV‐negative women. Most notably, in this study, HIV status was not significantly associated with any form of mistreatment (AOR: 0.83, 95% CI: 0.65–1.07), adjusted for age, education, marital status, and parity (Appiah et al. 2023).

Similarly, Sando et al. found that living with HIV in Tanzania was not significantly associated with any form of disrespect and abuse during childbirth, after adjusting for age, marital status, number of antenatal care visits for first pregnancy, employment, and parity (AOR: 0.83, 95% CI: 0.48–1.44) (Sando et al. 2014).

### 3.4 Physical Abuse

Physical abuse/violence occurred in approximately 2.7%–32.7% of cases. Sando et al. (2014) and Barabara et al. (2025) reported a prevalence of 2.7% (*n* = 4/147), and 14.6% (*n* = 15/103) in Tanzania respectively. Appiah et al. (2023) observed a prevalence of 15.5% (*n* = 48/310) in Ghana, and Assefa et al. reported a prevalence of 32.7% (*n* = 104/318) in Ethiopia (Assefa et al. 2022). An example of physical abuse was being administered an episiotomy without anesthesia (5.6%) (Sando et al. 2014). Further evidence revealed that women living with HIV in Tanzania had lower odds of physical abuse compared with HIV‐negative women, although this association was not significant (AOR: 0.72, 95% CI: 0.26–2.03) (Sando et al. 2014). On the other hand, Appiah et al. reported a significantly lower odds of physical abuse in Ghana among women living with HIV compared to HIV‐negative women (AOR: 0.50, 95% CI: 0.37–0.71) (Appiah et al. 2023). However, qualitative findings were corroborated by Barabara et al., who documented instances of physical abuse in Tanzania, where participants reported the threat of being slapped, and in some cases, actually slapped, if they “were not in the proper position” or “careless” during labor and delivery (Barabara et al. 2025).

### 3.5 Poor Rapport Between Women and Providers

Several women living with HIV reported negative interactions with their providers, which included non‐dignified care (e.g., verbal abuse), ineffective communication (e.g., failure to provide adequate information), and loss of autonomy (e.g., detention in healthcare facilities).

#### 3.5.1 Non‐Dignified Care

Women living with HIV frequently reported non‐dignified care in approximately 4.8% to 70.6% of cases across diverse regions (Sando et al. 2014; Appiah et al. 2023; Assefa et al. 2022; Barabara et al. 2025). Non‐dignified care was observed in Tanzania, Ghana, Ethiopia, India, the US, Malawi, South Africa, and Kenya (Jan et al. 2023; Appiah et al. 2023; Sethi et al. 2017; Cuca and Rose 2016; Weber et al. 2024; Gourlay et al. 2014; Onono et al. 2015; Assefa et al. 2022; Barabara et al. 2025). In some studies, verbal abuse was included as a component of non‐dignified care (Appiah et al. 2023; Sethi et al. 2017; Barabara et al. 2025).

Sethi et al. observed that women living with HIV in Malawi had greater odds of not being greeted respectfully (OR: 1.21, *p*: 0.79) and being yelled at, insulted, or threatened during labor (OR: 1.46, *p*: 0.53) compared to HIV‐negative women. However, these differences were not statistically significant (Sethi et al. 2017). Sando et al., and Barabara et al. observed a prevalence of 4.8% (*n* = 7/147) and 20.4% (*n* = 21/103) in Tanzania, respectively (Sando et al. 2014; Barabara et al. 2025). Sando et al. in their direct clinical observations noted that health care providers in Tanzania did not clean women living with HIV following delivery (17.7%, *n* = 3/18), often did not call them by their names throughout the visit (35.3%, *n* = 6/18), did not offer congratulatory remarks to them after delivery (47.1%, *n* = 8/18), and did not provide a clean bed in the postnatal ward (70.6%, *n* = 12/18) (Sando et al. 2014). Likewise, no significant differences in non‐dignified care were observed between women living with HIV and HIV‐negative women, except for not being called by their names (Sando et al. 2014). Further quantitative evidence revealed a prevalence of 21.3% (*n* = 66/310) in Ghana (Appiah et al. 2023), and 31% (*n* = 99/318) in Ethiopia (Assefa et al. 2022). Despite these findings, evidence from qualitative studies reveals that several women living with HIV experienced various forms of disrespect from their healthcare providers, including harsh and hostile treatment, verbal humiliation, and other forms of ill‐treatment during labor and delivery (Jan et al. 2023; Cuca and Rose 2016; Weber et al. 2024; Gourlay et al. 2014; Onono et al. 2015; Barabara et al. 2025).

#### 3.5.2 Ineffective Communication

Women living with HIV in South Africa, Kenya, Northern Ireland, Brazil, Vietnam, and Tanzania frequently reported that healthcare workers failed to provide adequate or consistent information during maternal care (Gourlay et al. 2014; Onono et al. 2015; Kelly et al. 2013; Malta et al. 2010; Nguyen et al. 2024; Barabara et al. 2025). In Tanzania, women living with HIV reported not being able to ask their providers questions (9.7%, *n* = 10/103), not understanding their providers' language (1.9%, *n* = 2/103), not receiving explanations for certain medical procedures (14.6%, *n* = 15/103), or medications (12.6%, *n* = 13/103) (Barabara et al. 2025).

Findings are supported by qualitative studies, which highlighted that women living with HIV often experience confusion when making critical decisions, such as when to initiate, stop, or continue breastfeeding their infants (Onono et al. 2015; Barabara et al. 2025), choose between cesarean section and spontaneous vaginal delivery (Kelly et al. 2013), due to conflicting or no guidance from healthcare providers. A study in Northern Ireland reported that women living with HIV were often confused by healthcare workers' perplexity regarding labor and delivery decisions, as some healthcare workers lacked the necessary skills in obstetric care (Kelly et al. 2013). Similarly, in Vietnam, some women living with HIV reported not receiving sufficient information on antiretroviral prophylaxis for their newborn after delivery, which resulted in their children getting infected with HIV (Nguyen et al. 2024). These knowledge gaps often leave women living with HIV uncertain about how to safely care for their infants, hindering opportunities for physical bonding due to unclear guidance (Nguyen et al. 2024).

#### 3.5.3 Loss of Autonomy

Women living with HIV reported loss of autonomy, which includes detention in a health facility, ranging from 0.7% (*n* = 1/147) in Tanzania (Sando et al. 2014) to 6.8% (*n* = 21/310) in Ghana (Appiah et al. 2023), lack of respect for women's birth preference (17.5%, *n* = 18/103) (Barabara et al. 2025), and being treated as passive participants (3.9%, *n* = 4/103) (Barabara et al. 2025) in Tanzania. Detention in healthcare facilities following delivery occurred due to the inability of some women living with HIV to pay their medical bills or provide the materials needed during childbirth (Barabara et al. 2025). Additionally, qualitative findings revealed that women living with HIV in Tanzania were often excluded from decisions regarding their health, which hindered their ability to communicate freely and make informed choices (Barabara et al. 2025). For instance, women living with HIV felt they could not express their desire for alternative birthing options with their providers (Barabara et al. 2025). However, in Malawi, women living with HIV had significantly lower odds of not being asked about their preferred position during delivery, compared to HIV‐negative women (OR: 0.17, 95% CI: 0.05–0.65) (Sethi et al. 2017).

### 3.6 Stigma and Discrimination

Fifteen studies highlighted encounters of stigma and discrimination among women living with HIV in healthcare settings because of their HIV status in the US, Uganda, Canada, Belgium, Tanzania, Kenya, Northern Ireland, Brazil, Peru, Vietnam, Cambodia, Nepal, Indonesia, and India (Jan et al. 2023; Cuca and Rose 2016; Ashaba et al. 2017; Greene et al. 2016; Arrey et al. 2017; Gourlay et al. 2014; Onono et al. 2015; Kelly et al. 2013; Valencia‐Garcia et al. 2017; Malta et al. 2010; Nguyen et al. 2024; Madhivanan et al. 2014; Lester et al. 1995; Women of the Asia Pacific Network of People Living with HIV 2012; Barabara et al. 2025). Appiah et al. observed that women living with HIV experienced more discrimination (13.5%, *n* = 42/310) compared to HIV‐negative women (10.8%, *n* = 198/1832) in Ghana (Appiah et al. 2023). Similarly, Lester et al. reported that 35% (*n* = 7/20) of women living with HIV experienced discrimination when interacting with their healthcare providers (Madhivanan et al. 2014). In this context, discrimination included inappropriate infectious disease control measures, such as displaying an “infectious precautions sign” on the patient's door (Madhivanan et al. 2014).

Further evidence from qualitative studies shows that women living with HIV were often criticized and stigmatized by their healthcare providers because of their desire to have children or being pregnant while HIV‐positive (Cuca and Rose 2016; Ashaba et al. 2017; Greene et al. 2016; Arrey et al. 2017; Gourlay et al. 2014; Lester et al. 1995; Women of the Asia Pacific Network of People Living with HIV 2012). Women living with HIV have sometimes been pressured to terminate their pregnancies (Cuca and Rose 2016; Greene et al. 2016; Malta et al. 2010; Lester et al. 1995), and were unjustly blamed for acquiring HIV due to sexual promiscuity (Arrey et al. 2017). In some Asian countries, 60% of women living with HIV reported that their abortion was a result of their HIV status (Women of the Asia Pacific Network of People Living with HIV 2012). Several women living with HIV in Vietnam indicated that the decision was influenced either by their assumptions or information they heard about their children becoming infected with HIV (Women of the Asia Pacific Network of People Living with HIV 2012). In addition, some healthcare professionals have actively discouraged women living with HIV from getting pregnant by creating a fear of having unhealthy babies or children who might become orphans (Greene et al. 2016; Women of the Asia Pacific Network of People Living with HIV 2012). In India, the birth of a child by a woman living with HIV has often been regarded as “divine punishment” by some healthcare providers (Jan et al. 2023).

Women living with HIV also reported differences in care and outward changes in behavior once physicians and nurses became aware of their status (Jan et al. 2023; Greene et al. 2016; Arrey et al. 2017). Healthcare workers often take actions, such as wearing multiple gloves without explaining the reason to the patient, keeping a physical distance, being extremely cautious around women living with HIV, and being treated as if they were not “normal” (Greene et al. 2016; Valencia‐Garcia et al. 2017; Lester et al. 1995; Women of the Asia Pacific Network of People Living with HIV 2012; Barabara et al. 2025).

### 3.7 Failure to Meet Professional Standards of Care

Evidence shows that healthcare providers sometimes fail to meet the standard of care. Specifically, women living with HIV frequently reported neglect and negligence, lack of informed consent and confidentiality, forced sterilization, non‐consensual child separation, and delayed access to quality healthcare. We discussed the specific unprofessional standard of care below.

#### 3.7.1 Forced Sterilization

Six studies documented findings on women living with HIV being pressured to undergo sterilization in maternal care settings (Bakare and Gentz 2020; Strode et al. 2012; Malta et al. 2010; Nguyen et al. 2024; Kendall and Albert 2015; Women of the Asia Pacific Network of People Living with HIV 2012). Experiences of forced sterilization were frequently reported in all four Latin American countries (Kendall and Albert 2015), South Africa (Strode et al. 2012), Vietnam (Nguyen et al. 2024), Namibia (Bakare and Gentz 2020), Brazil (Malta et al. 2010), and in some Asian countries (Cambodia, India, and Indonesia) (Women of the Asia Pacific Network of People Living with HIV 2012).

Findings from quantitative studies showed that forced sterilization or pressure to undergo sterilization were prevalent among women living with HIV, ranging from 17% to 38% (Kendall and Albert 2015; Women of the Asia Pacific Network of People Living with HIV 2012). Kendall and Albert (2015) reported experiences of forced sterilization among women living with HIV in four Latin American countries. Overall, 23% of women living with HIV were pressured to undergo sterilization after receiving their diagnosis (Kendall and Albert 2015). Among those who experienced the pressure to be sterilized, 17% of cases occurred in Nicaragua, 23% in El Salvador, 22% in Honduras, and 28% were reported in Mexico. Pregnant women living with HIV, in particular, were more likely to experience the pressure to be sterilized compared to their non‐pregnant counterparts (Kendall and Albert 2015). Pregnant women living with HIV had approximately six times the odds (AOR: 5.66, 95% CI: 2.35–13.58) of experiencing pressure to undergo sterilization (Kendall and Albert 2015). Kendall and Albert (2015) also reported that older women living with HIV between the ages of 25–34 (AOR: 0.18, CI: 0.04–0.91) and 45+ (AOR: 0.07, CI: 0.01–0.53) were less likely to be pressured to undergo sterilization compared to younger women living with HIV (≤ 24) (Kendall and Albert 2015).

A study conducted in five Asian countries reported that 38% (*n* = 86/228) of women living with HIV were not given the option to decline forced sterilization. Women living with HIV in India, Indonesia, and Cambodia (> 35%) reported the highest rate of being requested to undergo forced sterilization (Women of the Asia Pacific Network of People Living with HIV 2012). In this study, most Indonesian women living with HIV were more likely to be given an option to refuse the sterilization procedure (88.6%), compared to Cambodian women (48.6%) (Women of the Asia Pacific Network of People Living with HIV 2012). Also, 83% of the women living with HIV reported that sterilization was often recommended due to their HIV status, and usually came from their gynecologists and HIV providers (61.4%) (Women of the Asia Pacific Network of People Living with HIV 2012). Women living with HIV who delivered via cesarean section were more likely to be encouraged to undergo sterilization (43.5%) compared to those who had spontaneous vaginal delivery (30%) (Women of the Asia Pacific Network of People Living with HIV 2012). In addition, women living with HIV with more children, an average of two children, were more likely to be pressured to undergo sterilization compared to other women, although 4.6% of those pressured had no children. Age, maternal residence, and educational status were not associated with sterilization (Women of the Asia Pacific Network of People Living with HIV 2012).

Evidence from qualitative studies showed that women living with HIV were sterilized under certain conditions, such as preventing mother‐to‐infant HIV transmission and punishment for refusing to heed medical advice (Strode et al. 2012; Kendall and Albert 2015). For example, in some settings, sterilization was often presented as a necessary intervention to prevent mother‐to‐infant HIV transmission despite the availability of antiretroviral medications (ARVs) that could effectively reduce the risk of transmission (Strode et al. 2012; Malta et al. 2010; Nguyen et al. 2024; Kendall and Albert 2015). Women living with HIV have often reported that some healthcare workers viewed them as carriers of HIV and have stated that, without sterilization, their children could become infected or die (Kendall and Albert 2015). In addition, some healthcare providers used sterilization as a form of punishment for women living with HIV who became pregnant against medical advice or failed to follow their recommendations (Kendall and Albert 2015).

Some women living with HIV have complained of being sterilized without their informed consent and only found out after the procedures were done (Bakare and Gentz 2020; Women of the Asia Pacific Network of People Living with HIV 2012). For example, in Latin America, South Africa, and some Asian countries, women were sterilized either during a cesarean section or another type of surgery, and when they were under anesthesia (Strode et al. 2012; Kendall and Albert 2015; Women of the Asia Pacific Network of People Living with HIV 2012). Some women living with HIV reported signing consent forms for cesarean sections or other surgeries, but indicated that they were unaware they were also consenting to sterilization (Strode et al. 2012; Kendall and Albert 2015). In a particular case, healthcare providers were reported to have fabricated consent forms by making a mark of the patient's thumbprint during surgery, as a substitute for a signature (Kendall and Albert 2015). Some women living with HIV had no prior knowledge of being sterilized, discovering years later, either when attempting to get pregnant or during medical visits for a different health condition (Bakare and Gentz 2020; Strode et al. 2012). Other women living with HIV reported consenting to these procedures because they were not given adequate information about HIV from health care professionals and did not know how to exert their reproductive rights since they had no choice in the decision process (Bakare and Gentz 2020; Strode et al. 2012; Kendall and Albert 2015). Others have been compelled to accept sterilization to qualify for medical procedures and receive healthcare benefits such as cesarean delivery, financial support, and breast‐milk substitution (Bakare and Gentz 2020; Strode et al. 2012; Kendall and Albert 2015; Women of the Asia Pacific Network of People Living with HIV 2012).

#### 3.7.2 Lack of Non‐Confidential Care and Non‐Consented Care

Fifteen studies reported a lack of informed consent and breaches of confidentiality among women living with HIV (Jan et al. 2023; Sando et al. 2014; Appiah et al. 2023; Greene et al. 2016; Arrey et al. 2017; Weber et al. 2024; Kelly et al. 2013; Valencia‐Garcia et al. 2017; Nguyen et al. 2024; Madhivanan et al. 2014; Assefa et al. 2022; Lester et al. 1995; Women of the Asia Pacific Network of People Living with HIV 2012; Barabara et al. 2025). These findings have been commonly observed in several countries, including India, Tanzania, Ghana, Canada, Belgium, South Africa, Northern Ireland, Peru, Vietnam, Ethiopia, and the US.

Quantitative synthesis revealed that women living with HIV frequently experienced non‐consented care (1.4%–100%) (Sando et al. 2014; Appiah et al. 2023; Assefa et al. 2022; Barabara et al. 2025), and non‐confidential care (0.7%–33.2%) (Sando et al. 2014; Appiah et al. 2023; Barabara et al. 2025). Sando et al. reported an increased odds of non‐consented care among women living with HIV in Tanzania (AOR: 9.16, 95% CI: 1.73–115), adjusted for marital status, age, employment, and number of antenatal care visits during the first pregnancy (Sando et al. 2014). Also, although only one woman living with HIV reported non‐confidential care (0.7%, *n* = 1/147), and two reported non‐consented care (1.4%, *n* = 2/147) in childbirth, during direct clinical observations, Sando et al. observed that 22% (*n* = 4/18) of women living with HIV had their medical history frequently discussed in the presence of others. In addition, 88.9% (*n* = 16/18) were not asked for consent for their first examination in the antenatal ward, and none (100%, *n* = 18/18) were asked for consent prior to a vaginal examination during antenatal visits (Sando et al. 2014). However, only the lack of consent for vaginal examination differed significantly between women living with HIV and HIV‐negative women (*p* = 0.04) (Sando et al. 2014).

Likewise, Barabara et al. reported that 5.8% (*n* = 6/103) of women living with HIV in Tanzania did not have their medical records kept confidential, and 16.5% (*n* = 17/103) were not asked for consent before procedures were conducted (Barabara et al. 2025). Appiah et al. found that non‐confidential care occurred less frequently (33.2%, *n* = 103/310) among women living with HIV compared with HIV‐negative women (35.5%, *n* = 651/1832) in Ghana, with no significant difference between the groups. Similarly, non‐consented care was found to be prevalent among HIV‐negative women (7.6%, *n* = 139/1832) compared to women living with HIV (3.5%, *n* = 11/310) (Appiah et al. 2023).

Qualitative studies provided supportive evidence of non‐consented and non‐confidential care. Women living with HIV complained about how healthcare workers disclosed their diagnoses to other healthcare providers, family members, friends, and other patients without their consent (Jan et al. 2023; Greene et al. 2016; Arrey et al. 2017; Kelly et al. 2013; Valencia‐Garcia et al. 2017; Madhivanan et al. 2014; Lester et al. 1995; Women of the Asia Pacific Network of People Living with HIV 2012; Barabara et al. 2025). In India, the deliberate disclosure of HIV status by some healthcare workers has led to women living with HIV being ostracized by their family members and community (Madhivanan et al. 2014). Some also reported not consenting to medical procedures, for example, cesarean section (Weber et al. 2024; Nguyen et al. 2024; Barabara et al. 2025). Additionally, a study reported the unjust separation of the newborn from their mothers because of their HIV status after delivery, while leaving the mother unattended, and without obtaining informed consent and providing adequate explanation (non‐consensual child separation) (Weber et al. 2024).

The inability of some healthcare providers to maintain patients' rights to confidentiality and negative interactions encountered during maternal care have sometimes resulted in women living with HIV refusing to attend clinics, experiencing mental stress, rejecting further medical treatments for themselves or their babies, and resorting to self‐treatment (Weber et al. 2024; Gourlay et al. 2014; Valencia‐Garcia et al. 2017). In an extreme case, for example, a woman recounted being harshly confronted by a doctor who loudly disclosed her HIV status in a public waiting area. The experience left her feeling deeply humiliated and hopeless, to the point that she expressed a willingness to face death rather than endure such degrading treatment again (Valencia‐Garcia et al. 2017).

#### 3.7.3 Neglect and Abandonment, Delayed or Denied Access to Care, and Lack of Supportive Care

Fourteen studies reported neglect and abandonment, delayed or denied access to health care, and lack of supportive care among women living with HIV (Jan et al. 2023; Sando et al. 2014; Appiah et al. 2023; Sethi et al. 2017; Ashaba et al. 2017; Arrey et al. 2017; Weber et al. 2024; Onono et al. 2015; Valencia‐Garcia et al. 2017; Malta et al. 2010; Nguyen et al. 2024; Assefa et al. 2022; Women of the Asia Pacific Network of People Living with HIV 2012; Barabara et al. 2025). These findings were prevalent in Cambodia, Indonesia, Nepal, Vietnam, India, Tanzania, Ghana, Ethiopia, Uganda, Belgium, South Africa, Kenya, Peru, Brazil, and Malawi. Quantitative data showed that the prevalence of abandonment/neglect was 6.8% in Tanzania (Sando et al. 2014), 32.6% in Ghana (Appiah et al. 2023), and 44% in Ethiopia (Assefa et al. 2022).

The prevalence of lack of supportive care was 3.9%–28% (Women of the Asia Pacific Network of People Living with HIV 2012; Barabara et al. 2025). Examples of unsupportive care reported in Tanzania include healthcare providers being inattentive to their needs during labor and delivery (3.9%), failing to provide the best care (4.9%), not providing support for anxiety and fears (8.7%), not allowing patients to talk about their feelings with providers (15.5%), and prohibiting families and relatives from being available during labor (28%) or delivery (27%) (Barabara et al. 2025). Also, 33% of women living with HIV in Tanzania reported prolonged waiting times before receiving care (Barabara et al. 2025). Women living with HIV in Ethiopia (15.5%) also reported being denied admission to health facilities (Assefa et al. 2022).

Qualitative studies also reported that many women living with HIV experienced feelings of neglect and denied/delayed access to quality healthcare (Weber et al. 2024; Onono et al. 2015; Nguyen et al. 2024; Women of the Asia Pacific Network of People Living with HIV 2012), and in some instances some healthcare workers were afraid of touching them and contracting HIV (Women of the Asia Pacific Network of People Living with HIV 2012). Others have reported denial of medical services, such as fertility treatments, pain relief, and cesarean sections (Arrey et al. 2017; Valencia‐Garcia et al. 2017; Nguyen et al. 2024; Women of the Asia Pacific Network of People Living with HIV 2012; Barabara et al. 2025). In Vietnam, although women living with HIV reported that cesarean delivery was recommended by government policy for women living with HIV, many faced challenges in finding healthcare providers willing to perform the procedure due to fear of HIV transmission (Women of the Asia Pacific Network of People Living with HIV 2012). Another study in Vietnam reported that some women living with HIV were referred to other facilities because hospitals claimed they did not have the expertise in delivering their babies (Nguyen et al. 2024).

These negative experiences have resulted in women giving birth on their own and sometimes with the help of their family members, and without any support or care from healthcare providers (Weber et al. 2024; Onono et al. 2015; Nguyen et al. 2024). For instance, in a study, women living with HIV in South Africa reported delivering in non‐ideal conditions; one woman described having to deliver her child on the floor while simultaneously cleaning the birth fluids off the floor as instructed by the nurse, and another was repeatedly ignored during labor and was forced to “catch her baby.” (Weber et al. 2024). Furthermore, the study reported an account of a woman who experienced being neglected by nurses during her delivery, which may have contributed to her child sustaining a birth injury and requiring intensive care (Weber et al. 2024). In addition, some women living with HIV in Cambodia, Nepal, Indonesia, and Vietnam have reported waiting for an extended period, and are usually the last to be seen during clinical hours, even for procedures such as an ultrasound scan regardless of how early they arrive at the clinic (Women of the Asia Pacific Network of People Living with HIV 2012). Others have experienced being isolated from other patients in hospitals due to their HIV status (Greene et al. 2016; Valencia‐Garcia et al. 2017; Women of the Asia Pacific Network of People Living with HIV 2012).

### 3.8 Health System Conditions and Constraints

Health system constraints were often presented as a lack of resources (e.g., inadequate privacy, staff shortages, and insufficient medical supplies), as well as bribery and extortion by healthcare providers.

#### 3.8.1 Lack of Resources

Women living with HIV illustrated how maternal healthcare facilities failed to provide adequate resources, including medical supplies, physical privacy, and staff personnel. For example, in a study, women living with HIV in Tanzania frequently reported lack of a clean facility (10.7%), inadequate staffing (11.7%), unavailability of water (3.9%), and electricity in the facility (1.9%) (Barabara et al. 2025). In addition, women living with HIV have often complained of being reprimanded in front of other patients for failing to provide adequate personal protective equipment, such as gloves, during their care (Barabara et al. 2025). In another study, women living with HIV in South Africa described cases in which healthcare facilities were unable to provide necessary resources, such as ambulance transportation for emergency purposes and adequate bed space during care (Weber et al. 2024).

Women living with HIV also frequently reported a lack of privacy during maternal care (0.7%–94.4%) in Tanzania and Ethiopia (Sando et al. 2014; Assefa et al. 2022; Barabara et al. 2025). This can take various forms, including lack of visual privacy (29.1%, *n* = 30/103) (Barabara et al. 2025), being in a crowded space (38%, *n* = 39/103) (Barabara et al. 2025), not adequately covering the woman's naked body during labor and delivery (66.7%, *n* = 12/18), and providing partitions (94.4%, *n* = 17/18) (Sando et al. 2014). In contrast, during direct observations of labor and delivery, Sethi et al. observed that women living with HIV in Malawi were insignificantly less likely to be provided visual coverings to cover themselves during examinations and birthing procedures compared to HIV‐negative women (OR = 0.38, *p* = 0.23) (Sethi et al. 2017).

Women living with HIV also reported being requested to pay extra costs for maternal health services (Nguyen et al. 2024; Women of the Asia Pacific Network of People Living with HIV 2012). For instance, in some Asian countries, women reported being placed in private rooms during delivery and being mandated to pay the cost (Women of the Asia Pacific Network of People Living with HIV 2012). Additionally, women who wanted to have an abortion were often made to pay higher fees by healthcare providers for these services compared to HIV‐negative women (Women of the Asia Pacific Network of People Living with HIV 2012).

## 4 Discussion

This systematic review aimed to assess the prevalence and factors associated with the mistreatment of women living with HIV in maternal care settings. Our results showed evidence of a diverse range of mistreatments among women living with HIV. The overall prevalence of any form of obstetric mistreatment reported was 12.2% in Tanzania, 61% in Ghana, and 79% in Ethiopia (Sando et al. 2014; Appiah et al. 2023; Assefa et al. 2022). In this study, Sub‐Saharan Africa had the greatest burden of mistreatment experienced by women living with HIV, compared with other regions. Furthermore, the highest prevalence of individual forms of mistreatment across diverse regions was non‐consented care (1.4%–100%), lack of privacy (0.7%–94.4%), non‐dignified care (4.8%–70.6%), abandonment/neglect (6.8%–44%), discrimination/stigma (13.5%–35%), and forced sterilization (17%–38%).

Previous studies have examined the mistreatment of women during labor and childbirth in the general population and reported similar experiences ranging from 15% to 98% in various settings (Bohren et al. 2015; Gebeyehu et al. 2023; Abuya et al. 2018; USAID 2020; Okafor et al. 2015). A recent systematic review and meta‐analysis on the mistreatment of women in general during childbirth observed a global prevalence of 59% (Hakimi et al. 2025). Similar to their findings, non‐consented care was the most prevalent subcategory reported in our review. Another recent meta‐analysis reported a prevalence of obstetric mistreatment ranging from 5.6% to 91.7%, with higher prevalence in Africa (64.7%), the Eastern Mediterranean (50.9%), and the Americas (33.2%) (Özer et al. 2025). In Sub‐Saharan Africa, the overall prevalence was reported to be 44.09% (95% CI: 29.94–58.24) (Kassa et al. 2020). In Ethiopia, the pooled prevalence of mistreatment among women was 49.4% (95% CI: 30.9–68.1) (Kassa and Husen 2019). Although we were unable to conduct a meta‐analysis due to significant variation in the outcome assessment across studies, our results are comparable to those reported in the general population, suggesting a high burden of mistreatment among women living with HIV.

One possible explanation for the negative encounters women experience with healthcare providers often arises from systemic and structural barriers within healthcare systems. These include power dynamics that disproportionately affect women, poor work settings (e.g., inadequate staffing and medical resources, work overload, inadequate pay, long working hours) that could negatively impact healthcare providers, insufficient training or support for respectful maternal care, lack of accountability, and provider beliefs and biases (Mannava et al. 2015; Bohren et al. 2015; Bowser and Hill 2010; Schaaf et al. 2023; Khalil et al. 2025). For instance, in the US, 78.8% of women reported experiencing mistreatment during maternal care, especially when they had different opinions from their providers regarding their delivery (Vedam et al. 2019).

Furthermore, socio‐demographic factors have been identified as predictors of maltreatment in maternal health settings. In our review, we observed that women living with HIV who were older (≥ 35), with a primary education, and those whose newborns were delivered by a female birth attendant were more likely to be mistreated. Similar to our findings, Fernandes et al. in their review, reported that disadvantaged women in the general population (e.g., lack of education) in low‐ and middle‐income countries were at a higher risk of experiencing mistreatment from healthcare providers during childbirth (da Fernandes et al. 2023). Likewise, a study by the WHO in four countries (Nigeria, Ghana, Guinea, and Myanmar) reported a similar experience of mistreatment among younger (15–19 years) and less‐educated women in the general population (Bohren et al. 2019; World Health Organization (WHO) 2019). However, Hakim et al., in their systematic review, which focused on women in general, observed no significant differences in obstetric mistreatment between educated and uneducated mothers, and women younger or older than 19 years of age during maternal care (Hakimi et al. 2025).

### 4.1 Strengths and Limitations

This review focused on women living with HIV and the mistreatment they endure during pregnancy, labor, and postpartum, highlighting key issues. Another strength of our study is the use of a mixed‐methods synthesis, which allows for a comprehensive understanding of the experiences of women living with HIV in maternal healthcare by integrating quantitative and qualitative evidence and providing a holistic view of obstetric mistreatment. This systematic review has several limitations. First, most of the included studies were qualitative studies, which limits the generalizability of our findings. Second, some studies interviewed women years after they had given birth, which may increase the potential for recall bias. Third, the prevalence of different forms of obstetric mistreatment is inconsistent across studies and appears high in some instances; this finding should be interpreted with caution because there are few quantitative studies to draw inferences from. Lastly, we acknowledge the possibility of missing relevant publications during the systematic search due to the diverse and inconsistent terminology used to describe mistreatment, but our robust search and extensive hand search would have limited this.

## 5 Conclusions and Implications for Practice and Research

There is substantial evidence that women living with HIV continue to experience diverse forms of mistreatment in maternal care, particularly in Sub‐Saharan Africa. Women living with HIV were exposed to disrespectful maternal care practices, which included forced sterilization, lack of privacy, breaches of confidentiality and informed consent, non‐dignified care, and stigma and discrimination. Highlighting these disparities in care is crucial for improving continuous access to equitable healthcare services for women living with HIV. While most interventions have focused on reducing the mistreatment of women in general, a more tailored approach may be necessary for vulnerable populations, including women living with HIV, who may be at higher risk of experiencing obstetric mistreatment. As such, multicomponent interventions that include efforts to improve health system constraints, healthcare providers' behavior and attitudes, improving women's rights, and implementing supportive policy changes may likely enhance respectful maternal care among women living with HIV, especially in Sub‐Saharan Africa. Given the lack of consensus on the operationalization of obstetric mistreatment across studies, contributing to significant variability in reported prevalence rates, we recommend that future studies on obstetric mistreatment utilize standardized measurement tools. We also recommend using mixed‐methods studies for future research on mistreatment. Also, more studies should incorporate or utilize direct clinical observations when assessing mistreatment in maternal care to help bridge the gaps between reported and observed cases of mistreatment.

## Author Contributions

Conceptualization: Joy Edeh, Oluwaseun Badru, and Oluwafemi Adeagbo. Project administration: Joy Edeh and Oluwaseun Badru. Methodology: Joy Edeh, Oluwaseun Badru, Roba Alwasila, and Ezinwa Anyanwu. Data curation: Joy Edeh and Oluwaseun Badru. Formal analysis: Joy Edeh, Oluwaseun Badru, Roba Alwasila, and Ezinwa Anyanwu. Investigation: Joy Edeh, Oluwaseun Badru, Roba Alwasila, and Ezinwa Anyanwu. Writing – original draft: Joy Edeh and Oluwaseun Badru. Writing – review and editing: Joy Edeh, Oluwaseun Badru, Roba Alwasila, Ezinwa Anyanwu, and Oluwafemi Adeagbo. Visualization: Joy Edeh and Oluwaseun Badru. Validation: Joy Edeh, Oluwaseun Badru, Roba Alwasila, Ezinwa Anyanwu, and Oluwafemi Adeagbo. Supervision: Oluwaseun Badru and Oluwafemi Adeagbo.

## Funding

The authors have nothing to report.

## Ethics Statement

The authors have nothing to report.

## Conflicts of Interest

The authors declare no conflicts of interest.

## Supporting information


**Appendix S1:** The PRISMA checklist for this review.

**Appendix S2:** The full search strategy for all the databases.

**Appendix S3:** Representative quotes from the included studies.

**Appendix S4:** All items on the CASP qualitative appraisal tool and the score for each study.

## Data Availability

The data that support the findings of this study are available on request from the corresponding author. The data are not publicly available due to privacy or ethical restrictions.
