# Supplementary material for: A Systematic Review of Obstetric Mistreatment Among Women Living With HIV
Source: Nurs Health Sci. 2026 Mar 31;28(2):e70323. doi: 10.1111/nhs.70323 (PMC13038393; doi:10.1111/nhs.70323)
Supplement: Supplementary file 2 — Appendix S2: The full search strategy for all the databases. [file NHS-28-e70323-s002.docx]

**Supplementary Appendix S2: Search strategy for all databases**

PUBMED

| SN | Keywords | Hit |
| --- | --- | --- |
| 1 | **"obstetric violence" OR "obstetric mistreatment" OR mistreatment OR "disrespect and abuse" OR disrespect OR "humiliation and verbal abuse" OR "verbal abuse" OR "physical abuse" OR "non-consented care" OR "non-confidential care" OR "lack of privacy" OR "undignified care" OR abandonment OR "abandonment during labor" OR "abandonment during delivery" OR "abandonment of care" OR "abandonment after labor" OR "abandonment after delivery" OR "detention in facilit*" OR "discriminated care" OR "non-dignified care" OR "forced sterilization" OR "coerced sterilization" OR "unconsented medical procedure*" OR "violation of privacy" OR "refusal of admission" OR "physical violence" OR "neglected care" OR "microaggression" OR "negative birth experiences" OR "stigma and discrimination" OR stigma OR discrimination** | 463, 726 |
| 2 | **"women living with HIV" OR WLHIV OR WLWH OR WLWHA OR "HIV positive women" OR "HIV infected women"** | 8,721 |
| 3 | **("women living with HIV" OR WLHIV OR WLWH OR WLWHA OR "HIV positive women" OR "HIV infected women") AND ("obstetric violence" OR "obstetric mistreatment" OR mistreatment OR "disrespect and abuse" OR disrespect OR "humiliation and verbal abuse" OR "verbal abuse" OR "physical abuse" OR "non-consented care" OR "non-confidential care" OR "lack of privacy" OR "undignified care" OR abandonment OR "abandonment during labor" OR "abandonment during delivery" OR "abandonment of care" OR "abandonment after labor" OR "abandonment after delivery" OR "detention in facilit*" OR "discriminated care" OR "non-dignified care" OR "forced sterilization" OR "coerced sterilization" OR "unconsented medical procedure*" OR "violation of privacy" OR "refusal of admission" OR "physical violence" OR "neglected care" OR "microaggression" OR "negative birth experiences" OR "stigma and discrimination" OR stigma OR discrimination)** | 901 |

**WEB OF SCIENCE**

| SN | Keywords | Hit |
| --- | --- | --- |
| 1 | **"obstetric violence" OR "obstetric mistreatment" OR mistreatment OR "disrespect and abuse" OR disrespect OR "humiliation and verbal abuse" OR "verbal abuse" OR "physical abuse" OR "non-consented care" OR "non-confidential care" OR "lack of privacy" OR "undignified care" OR abandonment OR "abandonment during labor" OR "abandonment during delivery" OR "abandonment of care" OR "abandonment after labor" OR "abandonment after delivery" OR "detention in facilit*" OR "discriminated care" OR "non-dignified care" OR "forced sterilization" OR "coerced sterilization" OR "unconsented medical procedure*" OR "violation of privacy" OR "refusal of admission" OR "physical violence" OR "neglected care" OR "microaggression" OR "negative birth experiences" OR "stigma and discrimination" OR stigma OR discrimination** | 487,992 |
| 2 | **"women living with HIV" OR WLHIV OR WLWH OR WLWHA OR "HIV positive women" OR "HIV infected women"** | 9401 |
| 3 | S1 AND S2 | 1025 |

**SCOPUS**

| SN | Keywords | Hit |
| --- | --- | --- |
| 1 | TITLE-ABS-KEY ( "obstetric violence" OR "obstetric mistreatment" OR mistreatment OR "disrespect and abuse" OR disrespect OR "humiliation and verbal abuse" OR "verbal abuse" OR "physical abuse" OR "non-consented care" OR "non-confidential care" OR "lack of privacy" OR "undignified care" OR abandonment OR "abandonment during labor" OR "abandonment during delivery" OR "abandonment of care" OR "abandonment after labor" OR "abandonment after delivery" OR "detention in facilit*" OR "discriminated care" OR "non-dignified care" OR "forced sterilization" OR "coerced sterilization" OR "unconsented medical procedure*" OR "violation of privacy" OR "refusal of admission" OR "physical violence" OR "neglected care" OR "microaggression" OR "negative birth experiences" OR "stigma and discrimination" OR stigma OR discrimination ) | 564, 067 |
| 2 | TITLE-ABS-KEY ( "women living with HIV" OR wlhiv OR wlwh OR wlwha OR "HIV positive women" OR "HIV infected women" ) | 9, 980 |
| 3 | ( TITLE-ABS-KEY ( "obstetric violence" OR "obstetric mistreatment" OR mistreatment OR "disrespect and abuse" OR disrespect OR "humiliation and verbal abuse" OR "verbal abuse" OR "physical abuse" OR "non-consented care" OR "non-confidential care" OR "lack of privacy" OR "undignified care" OR abandonment OR "abandonment during labor" OR "abandonment during delivery" OR "abandonment of care" OR "abandonment after labor" OR "abandonment after delivery" OR "detention in facilit*" OR "discriminated care" OR "non-dignified care" OR "forced sterilization" OR "coerced sterilization" OR "unconsented medical procedure*" OR "violation of privacy" OR "refusal of admission" OR "physical violence" OR "neglected care" OR "microaggression" OR "negative birth experiences" OR "stigma and discrimination" OR stigma OR discrimination ) ) AND ( TITLE-ABS-KEY ( "women living with HIV" OR wlhiv OR wlwh OR wlwha OR "HIV positive women" OR "HIV infected women" ) ) | 1, 060 |

**CINAHL PLUS**

| SN | Keywords | Hit |
| --- | --- | --- |
| 1 | **"obstetric violence" OR "obstetric mistreatment" OR mistreatment OR "disrespect and abuse" OR disrespect OR "humiliation and verbal abuse" OR "verbal abuse" OR "physical abuse" OR "non-consented care" OR "non-confidential care" OR "lack of privacy" OR "undignified care" OR abandonment OR "abandonment during labor" OR "abandonment during delivery" OR "abandonment of care" OR "abandonment after labor" OR "abandonment after delivery" OR "detention in facilit*" OR "discriminated care" OR "non-dignified care" OR "forced sterilization" OR "coerced sterilization" OR "unconsented medical procedure*" OR "violation of privacy" OR "refusal of admission" OR "physical violence" OR "neglected care" OR "microaggression" OR "negative birth experiences" OR "stigma and discrimination" OR stigma OR discrimination** | 86, 739 |
| 2 | **"women living with HIV" OR WLHIV OR WLWH OR WLWHA OR "HIV positive women" OR "HIV infected women"** | 4,123 |
| 3 | S1 AND S2 | 608 |

EMBASE

| SN | Keywords | Hit |
| --- | --- | --- |
| 1 | **"obstetric violence" OR "obstetric mistreatment" OR mistreatment OR "disrespect and abuse" OR disrespect OR "humiliation and verbal abuse" OR "verbal abuse" OR "physical abuse" OR "non-consented care" OR "non-confidential care" OR "lack of privacy" OR "undignified care" OR abandonment OR "abandonment during labor" OR "abandonment during delivery" OR "abandonment of care" OR "abandonment after labor" OR "abandonment after delivery" OR "detention in facilit*" OR "discriminated care" OR "non-dignified care" OR "forced sterilization" OR "coerced sterilization" OR "unconsented medical procedure*" OR "violation of privacy" OR "refusal of admission" OR "physical violence" OR "neglected care" OR "microaggression" OR "negative birth experiences" OR "stigma and discrimination" OR stigma OR discrimination** | 345, 459 |
| 2 | **"women living with HIV" OR WLHIV OR WLWH OR WLWHA OR "HIV positive women" OR "HIV infected women"** | 10, 959 |
| 3 | #1 AND #2 | 1, 046 |

Google Scholar: obstetric violence, obstetric mistreatment, women living with HIV
