# Supplementary material for: A Systematic Review of Obstetric Mistreatment Among Women Living With HIV
Source: Nurs Health Sci. 2026 Mar 31;28(2):e70323. doi: 10.1111/nhs.70323 (PMC13038393; doi:10.1111/nhs.70323)
Supplement: Supplementary file 3 — Appendix S3: Representative quotes from the included studies. [file NHS-28-e70323-s004.docx]

**Supplementary Appendix S3: Representative** **Quotes from Qualitative Studies**

| **Themes** | **Representative Quotes** | **All contributing studies** |
| --- | --- | --- |
| **Physical abuse** | “If you are careless, they shout at you. They may tell you ‘Be in a proper position’; you can even be slapped; they may even slap you. They tell you don’t tighten your legs, that was their harshness.” (p. 5) (Barabara et al., 2025) | (Appiah, 2023; Assefa et al., 2022; Barabara et al., 2025; Sando et al., 2014) |
| **Stigma and discrimination** | “She (the nurse) called me like for an update. (I said) I’m like you know I’m gonna keep it because . . . my baby could be healthy. And she’s like are you sure? I’m like yes I’m sure. She’s like, well you only have a couple days to change your mind, are you sure you’re gonna to keep the baby? She asked me 10, 20 times and told me she was still gonna hold my spot for the abortion after I told her over and over again, I was keeping my baby.” (p. 6) (Greene et al., 2016)  “When I expressed my desire to have a child, my gynecologists sent me to the psychologist. I did not understand the reason because I was not depressed and knew exactly what I was asking for. I know that in Belgium women with HIV get pregnant and have children. I would have been less worried if the doctor had given me some reasons why I could not have a child.” (p. 9) (Arrey et al., 2017)  “The student doctor, once she found out I had HIV she doubled up on her gloves, she was terrified, cause I tore, to stitch me up . . . my nurses there were amazing I loved them all . . . those two ladies stood by me, hold hand when I cried, they hugged me and told me it’s okay . . . but the doctor and the um student doctor were the worst I’ve ever seen in my life. I will never recommend anyone to if a person has to go through that and have him, I will say screw it. Have your baby on the street before you have him.” (p. 10) (Greene et al., 2016)  “She [health worker] didn't help me ... because she was like, how can you get pregnant? Mmh, now you think you get pregnant without telling us. You are supposed to ask us instead of telling me that it has already happened... I tried explaining to her but she kept laughing, I just kept quiet.” (p. 6) (Ashaba et al., 2017) | (Arrey et al., 2017; Ashaba et al., 2017; Barabara et al., 2023; Cuca & Rose, 2016; Gourlay et al., 2014; Greene et al., 2016; Jan et al., 2023; Kelly et al., 2013; Lester et al., 1995; Madhivanan et al., 2014; Malta et al., 2010; Nguyen et al., 2024; Onono et al., 2015; Valencia-Garcia et al., 2017) |
| **Poor rapport between women and providers** |  |  |
| **Non-dignified care** | “The nurse who instructed me to step down from the bed shouted at me wanted the reason for giving birth (likely mucous or fluids) on the floor. I replied to her that she is the one who instructed me to step down from the bed and I had to follow her instructions as I was afraid of her. The floor was dirty, and she gave me something to clean the floor. […] She said, ‘I can’t work on a dirty area; you must clean the floor because there are no cleaners over the night.’ I started to clean, and I could still feel that my baby was coming. I started by cleaning where the nurse was going to walk pass[ed] and my water broke (likely mucous or fluids) while I was busy cleaning. I ended up giving birth to my baby while I was standing on the floor.” (p. 6) (Weber et al., 2024)  “….a nurse instructed me to catch my baby. She [the nurse] said, ‘Who has to catch your baby if you don’t?” The ward was dirty by then. She said, ‘What must I do and how could I attend to you when the ward is dirty like this?’ I said, ‘I told you from the beginning that my baby is coming; you were supposed to attend to me long time ago’ She said, ‘Stay there; I will take your baby and I will leave you dirty like that until I have time to attend to you.’” (p. 6) (Weber et al., 2024)  “Well, you know, if God was here, he’d probably strike you to hell already” . . . . And it devastated me. After I went home I sat, I sat down and all I could do was just cry. I cried, I cried, I cried, I cried, I cried, I cried.” (p. 5) (Cuca & Rose, 2016)  “When I went to the hospital I met a certain lady who was so harsh to me. When I told her it was time she chased me away and told me it was not yet. When I was out on the veranda I felt the baby coming and I delivered my baby. That is why am saying that here at the hospital they don’t treat us well.” (p. 5) (Onono et al., 2015) | (Appiah, 2023; Assefa et al., 2022; Barabara et al., 2025; Gourlay et al., 2014; Jan et al., 2023; Onono et al., 2015; Sando et al., 2014; Sethi et al., 2017) |
| **Ineffective communication** | “We were the blind leading the blind. None of us knew anything about it [….]I had all these questions and all I thought was I am going to die and that is it. I will have to get rid of the baby and I knew nothing. They knew nothing [raises voice]. They couldn’t tell me anything because they didn’t know [….] Just make sure that you are told in probably the appropriate place with people who actually know and can explain every thing to you.” (p. 4) (Kelly et al., 2013)  “The only small problem was that they were giving different information. In clinic the girls were telling me ‘no it can’t be a natural birth, you must be sectioned’. […] They are supposed to consult together and not to confuse people too much. That was the only thing that was making me to lose some confidence […]. Finally, to be honest, almost until I went to give birth, I wasn’t sure it would be naturally or it would be section. Until that stage I was still confused.” (p. 4) (Kelly et al., 2013)  “It was after a month when I came to get her examinations done that the nurse asked me whether my daughter was taking medications. I told her that I didn’t know…The staff over at the hospital said that as soon as we went home after the birth, they suddenly remembered us. They said that I did not pick up the phone when they called. But no one was calling me. Those nurses said it was my fault!.” (p. 5) (Nguyen et al., 2024)  “They helped me deliver, and that’s all. There were no medications. There was nothing here back then. My baby got infected. After that time, I didn’t dare give birth. To date, I still have not given birth again.” (p. 5) (Nguyen et al., 2024) | (Barabara et al., 2025; Gourlay et al., 2014; Kelly et al., 2013; Malta et al., 2010; Nguyen et al., 2024; Onono et al., 2015) |
| **Loss of autonomy** | “Nurses do not allow you to stand during labour and delivery. They advise you to lie on the bed when you feel the urge to push. They may explain that it is not safe to squat or sit down during this stage of labour and delivery.” (p. 7) (Barabara et al., 2025) | (Appiah, 2023; Barabara et al., 2025; Sando et al., 2014) |
| **Failure to meet professional standards of care** |  |  |
| **Forced sterilization** | ‘‘I was in labor, and what I wanted was to receive care. Dr. [X] really pressured me to accept sterilization, saying, ‘What kind of life are you going to give to your child?” (p. 4) (Kendall & Albert, 2015)  “I started taking the medicine that helped prevent HIV transmission to my baby. When I had my delivery…the doctors made me undergo sterilization. I accepted…I was afraid that my daughter would be infected with HIV, so I accepted the operation.” (p. 4) (Nguyen et al., 2024)  “I went to the hospital to give birth in 2007, I went there on the 7th of December, I was told that I am going to the theatre, but I was not told that by going to the theatre I am going to be sterilised also, I was just told that you are going to have surgery because you are living with HIV we just want to help your baby not get infected by the virus, you are just going to be operated so that we can remove the baby, nothing else is going to be done … It was only in 2013 when I found out because of the sterilisation campaign … I went to the office of Namibia Health Women Network, they saw on my health passport that I am sterilized.” (p. 5-6) (Bakare & Gentz, 2020)  “He [the doctor] asks how many kids I have. I say it’s the second one. Do I have any knowledge about how risky it would be for me to get another child being HIV-positive?... Then he said the way he sees it I must be sterilized because it’s a risk.” (p. 5 ) (Strode et al., 2012) | (Bakare & Gentz, 2020; Kendall & Albert, 2015; Malta et al., 2010; Nguyen et al., 2024; Strode et al., 2012; Women of the Asia Pacific Network of People Living with HIV, 2012) |
| **Lack of non-confidential care and non-consented care** | “Midwife’s name] went and told three or four different mid wives and I never consented to any of them knowing. They never had to deal with me, see me. They didn’t know me like. You know I would never let any of them ladies, like, examine me and never know or take bloods and never know, nothing like that there like. You know what I mean. But they had no right to just go and tell.” (p. 5) (Kelly et al., 2013)  “A woman [the wife of my husband’s friend] noticed that I was not breast-feeding my baby. She asked me why and I told her that I did not have enough breast milk. She was not satisfied with my response. She later informed me that she had called the maternity unit where I had my baby to inquire about me and was told that I have HIV. My husband and I were very angry and had contemplated taking legal action against the clinic for unlawful disclosure. We did not pursue with legal action because we did not want more people to know my status.” (p. 10 ) (Arrey et al., 2017)  “Everybody heard what I was told (by providers): ‘You are coming here without gloves, and you know your status.’ People must have understood what was going on (my HIV status), so you will feel ashamed…... I felt embarrassed, I felt bad, I wished I could give birth on that day and be discharged on the very same day.” (p. 13) (Barabara et al., 2025)  “I never wanted it (cesarean section) either. But they said the baby’s head circumference was too big. […] They never told me anything, they just sent me to the theatre because they didn’t want to risk and keep me waiting while I had already waited for a very long time.” (p. 7) (Weber et al., 2024) | (Appiah, 2023; Arrey et al., 2017; Assefa et al., 2022; Barabara et al., 2023, 2025; Greene et al., 2016; Jan et al., 2023; Kelly et al., 2013; Lester et al., 1995; Madhivanan et al., 2014; Nguyen et al., 2024; Sando et al., 2014; Valencia-Garcia et al., 2017; Weber et al., 2024; Women of the Asia Pacific Network of People Living with HIV, 2012) |
| **Neglect and Negligence/Delayed or denied access to care**  **Lack of Supportive care** | “When I entered the delivery room, the doctors there discriminated against me. The doctors didn’t want to take care of me. There were many times when I had labor pain and I asked for an operation multiple times, but the doctors just ignored me.” (p. 4) (Nguyen et al., 2024)  “They [the hospital staff] didn’t dare to touch me. It was in 2006 when people didn’t know much about my illness…At that time, while I was in pain, they told me to keep waiting, waiting, waiting... I gave birth right on the spot. My husband delivered my baby…no one would help me.” (p. 4) (Nguyen et al., 2024)  “I stood up and I felt something coming out of my vagina…it was the head of the baby. I screamed immediately shouting and calling nurses. The other nurse responded by saying “we are not your kids come to us”. I told them that I cannot move or even raise my leg because I was afraid the baby might fall, they ignored me and continue with their conversation. I had to drag my feet to the bed, and I could feel the head of the baby was stuck in the vaginal opening. When I get to the bed, they said I should get off that bed and go to the other bed. I had to drag my feet getting to the other bed….it was this time that they realized that this was something serious they came (nurses) and assisted me to deliver. I just pushed three times and the baby came out.”  “What was shocking is that my baby didn’t cry and the head of my baby including the face turned purple because the blood was not circulating while the head was stuck in the vaginal opening, she had internal bleeding even the (baby’s) mouth was purple. […] This is a very serious case of negligence because it is not as if the nurses were busy with something else, but they were just seating on the table chatting. It was very serious, and they even said themselves that I almost lost my baby.” (p. 7) (Weber et al., 2024)  “I was nine months pregnant and felt my baby was about to come. I went to the maternity unit with my husband, and I was sent back home despite the fact that it was my first pregnancy, and I was in much pain. Without examining me the nurses at the maternity unit asked my husband to take me back home and come back after two or three days. We went back home and immediately my waters broke and I started pushing very hard. My husband had no time to call for the ambulance. He drove me back to the hospital and I was rushed to the delivery room. I had my baby about ten minutes after I arrived. I still have pains in my lower back and I believe they originated from the unattended labor.” (p.9) (Arrey et al., 2017)  “When I was pregnant they [hospital staff] treated me very bad. It was a bad experience [sobbing]. They didn’t want me to shower, they didn’t want anyone to enter my room, they didn’t want to hold my daughter, they would come in upset and grab my daughter to change her. Because I had a cesarean section they didn’t want to tend to my wound, they would come in a bad mood and against their will, you could feel it and see it, and that would make me more depressed.”  (p.6) (Valencia-Garcia et al., 2017)  “When I went into labour (her previous pregnancy), they gave me the bed, but they were hesitating to examine me. The pain was greater, I called, but they did not come, later when she examined me, she said ‘You’re very dilated. What were you waiting for there?” (p. 6) (Barabara et al., 2025)  “[The midwife] was too busy with their telephone, using ear- phones…Yes. I can say that maybe they did not hear me.” (p. 6) (Barabara et al., 2025)  “It is not allowed. I mean if your relatives should come, they stop at the door, they don’t enter. The nurses will be very angry and upset if they should find a relative in there.”  (p. 6) Barabara et al.(Barabara et al., 2025)  “I would have appreciated having my mother by my side.”  (p. 6) Barabara et al.(Barabara et al., 2025) | (Appiah, 2023; Arrey et al., 2017; Ashaba et al., 2017; Assefa et al., 2022; Barabara et al., 2025; Jan et al., 2023; Malta et al., 2010; Nguyen et al., 2024; Onono et al., 2015; Sando et al., 2014; Sethi et al., 2017; Valencia-Garcia et al., 2017; Weber et al., 2024; Women of the Asia Pacific Network of People Living with HIV, 2012) |
| **Health system conditions and constraints** |  |  |
| **Lack of resources** | “Most people knew that I got HIV so they wouldn’t bathe the baby. We just bathed our baby ourselves. Also, in that hospital, there were no wound care services…my family asked, they just said no.” (p. 5) (Nguyen et al., 2024)  “because there were no ambulances available…my baby had to leave first, and I followed a bit later.” (p.5) (Weber et al., 2024)  “I was told to wait because the beds were occupied. I was then admitted later on. (p.5) (Weber et al., 2024)  “informed that there were no beds available for me, I slept in the passages for days.” (p.5) (Weber et al., 2024) | (Barabara et al., 2023; Nguyen et al., 2024; Weber et al., 2024; Women of the Asia Pacific Network of People Living with HIV, 2012) |
| **Bribery/Extortion** | “When it came to my operation, the woman who did my hospital papers said that I had to pay more to support the doctors, the operation team. But I saw that my friends… for this same thing...they paid 1 million dong, some 2 million, and some 3 million… but for me the deal was 7 million dong. 7 million is not a big amount of money, but for my husband and I, it was everything. There were more payments with paper signing, in total it was 11 million dong or so.” (p. 5) (Nguyen et al., 2024) | (Gourlay et al., 2014; Nguyen et al., 2024; Women of the Asia Pacific Network of People Living with HIV, 2012) |
