# Supplementary material for: A Systematic Review of Obstetric Mistreatment Among Women Living With HIV
Source: Nurs Health Sci. 2026 Mar 31;28(2):e70323. doi: 10.1111/nhs.70323 (PMC13038393; doi:10.1111/nhs.70323)
Supplement: Supplementary file 4 — Appendix S4: All items on the CASP qualitative appraisal tool and the score for each study. [file NHS-28-e70323-s003.docx]

**Supplementary Appendix S4: Quality appraisal of included studies**

**Qualitative Studies assessed using Critical Appraisal Skills Program (CASP) checklist (n = 15)**

| Study Type | Qualitative Questions | | | | | | | | | | | | |
| --- | --- | --- | --- | --- | --- | --- | --- | --- | --- | --- | --- | --- | --- |
|  | 1 | 2 | 3 |  | 4 | 5 | 6 | 7 | 8 | 9 | 10 |  | **Comments** |
| Qualitative | Was there a clear statement of the aims of the research?  2  2  2  2  2  2  2  2  2  2  2 | Is a qualitative methodology appropriate?  2  2  2  2  2  2  2  2  2  2  2 | Is it worth continuing  Yes Yes Yes Yes Yes Yes Yes Yes Yes Yes Yes | Was the research design appropriat e to address the aim of research?  2  2  2  2  2  2  2  2  2  2  2 | Was the recruitme nt strategy appropriat e to the aims of the research?  2  2  2  0  2  2  2  2  2  2  2 | Was the data collected in a way that addresse d the research issue?  2  2  2  1  2  2  2  2  2  2  2 | Has the relationship between researcher and participants been adequately considered?  0  0  0  0  0  0  0  0  0  0  0 | Have | Was the data analysis sufficiently rigorous?  2  2  1  1  2  2  2  2  2  2  2 | Is there a clear statement of findings?  2  2  2  1  2  2  2  2  2  2  2 | How valuable is the research?  2  2  2  1  2  2  2  2  2  2  2 | **Total** |  |
| study |  |  |  |  |  |  |  | ethical |  |  |  | **score** |  |
|  |  |  |  |  |  |  |  | issues |  |  |  |  |  |
| Citation |  |  |  |  |  |  |  | been |  |  |  |  |  |
| (Evaluation |  |  |  |  |  |  |  | taken |  |  |  |  |  |
| sample size) |  |  |  |  |  |  |  | into |  |  |  |  |  |
|  |  |  |  |  |  |  |  | conside |  |  |  |  |  |
|  |  |  |  |  |  |  |  | ration? |  |  |  |  |  |
| Greene et |  |  |  |  |  |  |  | 2 |  |  |  | **18** | **Moderate** |
| al.2016 |  |  |  |  |  |  |  |  |  |  |  |  |  |
| Arrey et al. |  |  |  |  |  |  |  | 2 |  |  |  | **18** | **Moderate** |
| 2016 |  |  |  |  |  |  |  |  |  |  |  |  |  |
| Bakare et |  |  |  |  |  |  |  | 2 |  |  |  | **17** | **Moderate** |
| al.2020 |  |  |  |  |  |  |  |  |  |  |  |  |  |
| Jan et al. |  |  |  |  |  |  |  | 1 |  |  |  | **11** | **Low** |
| 2023 |  |  |  |  |  |  |  |  |  |  |  |  |  |
| Weber et al. |  |  |  |  |  |  |  | 2 |  |  |  | **18** | **Moderate** |
| 2024 |  |  |  |  |  |  |  |  |  |  |  |  |  |
| Strode et al. |  |  |  |  |  |  |  | 2 |  |  |  | **18** | **Moderate** |
| 2012 |  |  |  |  |  |  |  |  |  |  |  |  |  |
| Gourlay et al. |  |  |  |  |  |  |  | 2 |  |  |  | **18** | **Moderate** |
| 2014 |  |  |  |  |  |  |  |  |  |  |  |  |  |
| Onono et al. |  |  |  |  |  |  |  | 2 |  |  |  | **18** | **Moderate** |
| 2015 |  |  |  |  |  |  |  |  |  |  |  |  |  |
| Kelly et al. |  |  |  |  |  |  |  | 2 |  |  |  | **18** | **Moderate** |
| 2013 |  |  |  |  |  |  |  |  |  |  |  |  |  |
| Malta et al. |  |  |  |  |  |  |  | 2 |  |  |  | **18** | **Moderate** |
| 2010 |  |  |  |  |  |  |  |  |  |  |  |  |  |
| Asaba et al. |  |  |  |  |  |  |  | 2 |  |  |  | **18** | **Moderate** |

| 2017 |  |  |  |  |  |  |  |  |  |  |  |  |  |
| --- | --- | --- | --- | --- | --- | --- | --- | --- | --- | --- | --- | --- | --- |
| Cuca et al. |  |  |  |  |  |  |  |  |  |  |  |  |  |
| 2016 | 2 | 2 | Yes | 2 | 2 | 2 | 0 | 2 | 2 | 2 | 2 | **18** | **Moderate** |
| Nguyen et al. |  |  |  |  |  |  |  |  |  |  |  |  |  |
| 2024 | 2 | 2 | Yes | 2 | 2 | 2 | 0 | 2 | 1 | 2 | 2 | **17** | **Moderate** |
| Madhivanian |  |  |  |  |  |  |  |  |  |  |  |  |  |
| et al. 2014 | 2 | 2 | Yes | 2 | 2 | 2 | 0 | 2 | 2 | 2 | 2 | **18** | **Moderate** |
| Garcia et al. |  |  |  |  |  |  |  |  |  |  |  |  |  |
| 2017 | 2 | 2 | Yes | 2 | 2 | 2 | 0 | 2 | 2 | 2 | 2 | **18** | **Moderate** |
|  |  |  |  |  |  |  |  |  |  |  |  |  |  |

**2 = criterion fully met, 1 = partially met, and 0 = not met**

**Quantitative Studies assessed using the Joanna Briggs Institute (JBI) critical appraisal checklist (n = 3)**.

| Study Type | Quantitative Questions | | | | | | | | |
| --- | --- | --- | --- | --- | --- | --- | --- | --- | --- |
|  | 1 | 2 | 3 | 4 | 5 | 6 | 7 | 8 |  |
| Quantitative Study | Were the criteria for inclusion in the sample clearly defined? | Were the study subjects and the setting described in detail? | Was the exposure measured in a valid and reliable way? | Were objective, standard criteria used for measurement of the condition?  Yes  Yes  Yes | Were the confounding factors identified?  Yes  Yes  No | Were the strategies to deal with confounding factors stated?  Yes  Yes  No | Were the outcomes measured in a valid and reliable way? | Was appropriate statistical analysis used?  Yes  Yes  Yes | Comment |
| Appiah et al. 2023 | Yes | Yes | Yes |  |  |  | Yes |  | 8/8: High quality |
| Assefa et al. 2022 | Yes | Yes | Yes |  |  |  | Yes |  | 8/8: High quality |
| Sethi et al. 2017 | Yes | Yes | Yes |  |  |  | Yes |  | 6/8: Moderate quality |
|  |  |  |  |  |  |  |  |  |  |

**A score of 0 - 3 is considered low quality, 4 - 6 is moderate quality, and 7 - 8 is high quality**

**Mixed Method Studies assessed using the Mixed Method Appraisal tool (MMAT) (n = 5)**

| Study Type | Mixed Method Questions | | | | | | |  |
| --- | --- | --- | --- | --- | --- | --- | --- | --- |
|  | 1 | 2 | 3 | 4 | 5 | 6 | 7 |  |
| Mixed Method study  Citation (Evaluation sample size) | Are there clear research questions?  Yes  Yes  Yes  Yes  Yes | Do the collected data allow to address the research questions?  Yes  Yes  Yes  Yes  Yes | Is there an adequate rationale for using a mixed methods design to address the research question?  Yes  Yes  Yes  Yes  Yes | Are the different components of the study effectively integrated to answer the research question?  Yes  Yes  Yes  Yes  Yes | Are the outputs of the integration of qualitative and quantitative components adequately interpreted?  Yes  Yes  Yes  Yes  Yes | Are divergences and inconsistencies between quantitative and qualitative results adequately addressed?  Yes  Can’t tell  Yes  Yes  Can’t tell | Do the different components of the study adhere to the quality criteria of each tradition of the methods involved? | Comment |
| Sando et al.2014 |  |  |  |  |  |  | Yes | 7/7: High quality |
| kendal et al.2015 |  |  |  |  |  |  | Yes | 6/7: High quality |
| Lester et al.1995 |  |  |  |  |  |  | Yes | 7/7: High quality |
| Barabara et al.2023 |  |  |  |  |  |  | Yes | 7/7: High quality |
| Women of the Asia Pacific Network of People Living with HIV. 2012 |  |  |  |  |  |  | Can’t tell | 5/7: High quality |

**Scores of ≥ 6: high quality, 3 – 5 were: moderate quality, and ≤ 2: low quality**
